# Profiling transcriptome composition and dynamics within nuclear compartments using SLAM-RT&Tag

**DOI:** 10.1016/j.molcel.2025.02.012

**Published:** 2025-03-11

**Authors:** Nadiya Khyzha, Kami Ahmad, Steven Henikoff

**Affiliations:** 1 Basic Sciences Division, Fred Hutchinson Cancer Research Center; Seattle, WA, 98109, USA; 2 Howard Hughes Medical Institute; Chevy Chase, MD, 20815, USA; 3 Lead contact

## Abstract

Nuclear compartments are membrane-less regions enriched in functionally related molecules. RNA is a major component of many nuclear compartments, but the identity and dynamics of transcripts within nuclear compartments are poorly understood. Here, we applied Reverse Transcribe & Tagment (RT&Tag) to human cell lines to identify the transcript populations of Polycomb domains and nuclear speckles. We also developed SLAM-RT&Tag, which combines RNA metabolic labeling with RT&Tag, to quantify transcript dynamics within nuclear compartments. We observed unique transcript populations with differing structures and dynamics within each compartment. Intriguingly, exceptionally long genes are transcribed adjacent to Polycomb domains, and are transiently associated with chromatin. In contrast, nuclear speckles act as quality control checkpoints that transiently confine incompletely spliced polyadenylated transcripts and facilitate their post-transcriptional splicing. In summary, we demonstrate that transcripts at Polycomb domains and nuclear speckles undergo distinct RNA processing mechanisms, highlighting the pivotal role of compartmentalization in RNA maturation.

## Graphical Abstract

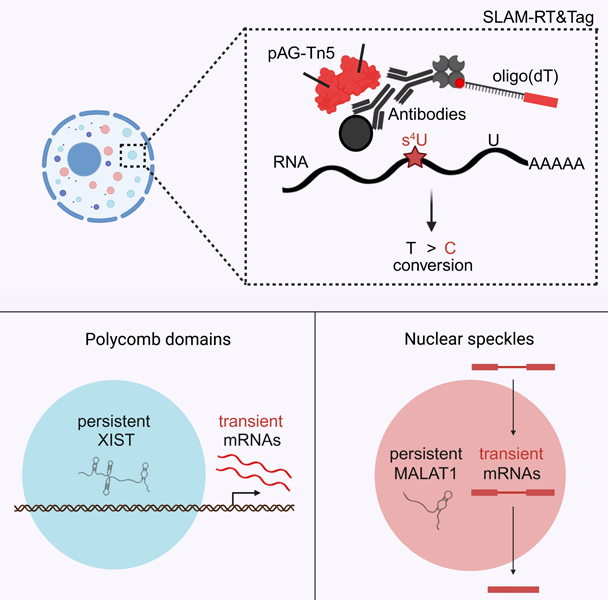

## Introduction

The nucleus contains numerous membrane-less compartments such as nuclear structures, bodies, and chromatin domains^1^. These nuclear compartments are broadly defined as regions enriched in functionally related molecules, and the high concentrations of specific proteins or nucleic acids may promote biological processes^2^. RNA is an integral component of many nuclear compartments whereby it can trigger compartment formation and be processed within these compartments^3^. Thus, identifying RNAs localized within nuclear compartments and assessing their dynamics is crucial for understanding the mechanisms taking place within these compartments.

Chromatin domains can compact into distinct chromosome structures, thus forming compartments^1^. For example, the inactive X chromosome in mammals is marked by the inactive H3K27me3 histone mark and forms a condensed structure called the Barr body^4^. The establishment of the Barr body is orchestrated by the long noncoding RNA (lncRNA), *XIST,* which is stably associated with the inactive X chromosome^3^. Polycomb domains are likewise transcriptionally silenced H3K27me3-marked chromatin domains^5^. Polycomb domains preferentially contact each other in 3D nuclear space^6,7^ and form discrete foci that can be observed cytologically^8^. It has been debated whether RNA is implicated in the regulation of Polycomb domains in mammals^9,10^. Whether RNA is present within Polycomb domains and whether it is stably associated with them remains to be determined.

Nuclear speckles are nuclear bodies containing large amounts of RNA processing factors (particularly splicing factors), and RNA (lncRNA *MALAT1* and polyadenylated mRNAs)^11–13^. Multiple functions are associated with nuclear speckles including being storage sites, regulation of splicing factor concentration in the nucleoplasm, splicing efficiency, and amplification of transcription^14^. Additionally, RNA fluorescence *in situ* hybridization (RNA-FISH) and RNA reporter assays showed that some transcripts migrate to nuclear speckles to be spliced or to gain nuclear export competence^15–20^. Further work is needed to elucidate the broader trends in mechanisms driving endogenous RNA migration to nuclear speckles and the dynamics of transcripts within nuclear speckles.

Here, we apply our Reverse Transcribe and Tagment (RT&Tag) method^21^ to profile the transcriptome of two key nuclear compartments, Polycomb domains and nuclear speckles. These compartments were chosen due to their differences in transcriptional activity and splicing factor concentrations^22^. Additionally, we developed SLAM-RT&Tag, which applies RNA metabolic labeling to RT&Tag^23^, to follow the dynamics of transcripts within nuclear compartments. Using RT&Tag and SLAM-RT&Tag, we identified transcript populations with distinct structures and dynamics within the two compartments. We did not detect transcription within Polycomb domains. Instead, we detected transcripts originating from long genes that are transcribed adjacent to Polycomb domains. In the case of nuclear speckles, we propose that they function as splicing quality control checkpoints that incompletely spliced polyadenylated transcripts migrate through to undergo post-transcriptional splicing. Overall, we demonstrate that transcripts at Polycomb domains and nuclear speckles have separate RNA processing mechanisms, underscoring the role of compartmentalization in RNA maturation.

## Results

### RT&Tag detects RNA within nuclear compartments.

**Figure 1:**
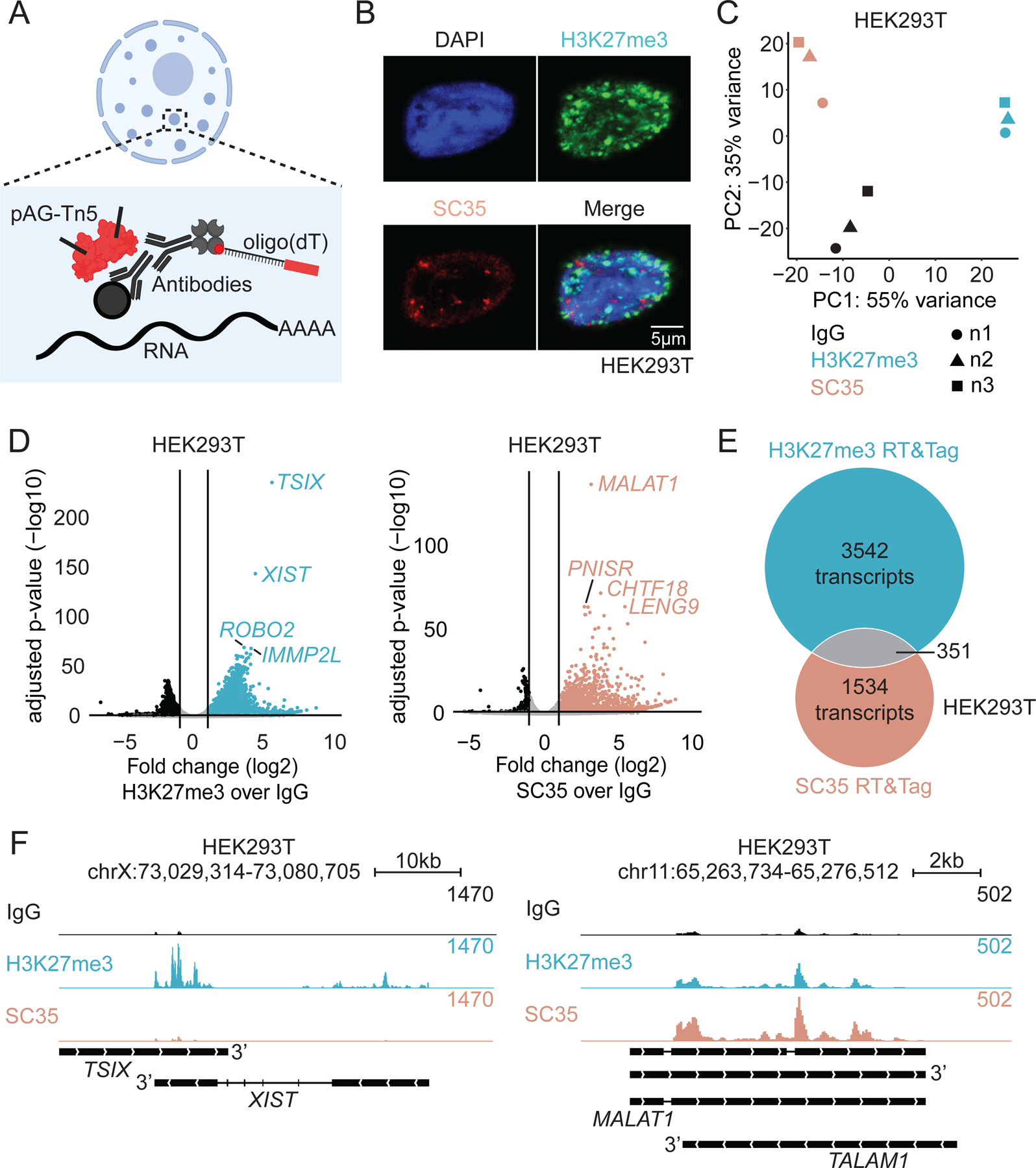
RT&Tag detects RNA within nuclear compartments. A) RT&Tag schematic: antibodies targeting a nuclear compartment epitope are used to tether pAG-Tn5 and an oligo(dT). B) Immunofluorescence of HEK293T nuclei stained for DAPI, H3K27me3 and SC35. Scale bar, 5μm. C) Principal component analysis of IgG, H3K27me3, and SC35-targeted RT&Tag in HEK293T cells. D) Volcano plot showing transcripts differentially enriched for H3K27me3 (left) and SC35 (right) over IgG-targeted RT&Tag in HEK293T cells (log_2_ FC>2, FDR<0.05, n=3). E) Overlap of transcripts enriched for H3K27me3 and SC35-targeted RT&Tag in HEK293T cells. F) Genome tracks showing IgG, H3K27me3, and SC35-targeted RT&Tag signal over *XIST* (left) and *MALAT1* (right) in HEK293T cells. See also Figure S1.

We applied the Reverse Transcribe & Tagment (RT&Tag) method^21^ to capture transcripts within Polycomb domains and nuclear speckles. RT&Tag is an *in situ* antibody-mediated enzyme tethering method that uses antibodies to tether an oligo(dT) primer and a Tn5 enzyme to an epitope of interest (Figure 1A). RNA near the epitope is then reverse transcribed, RNA-cDNA hybrids are tagmented by the Tn5 enzyme, and Illumina sequencing libraries are generated using PCR. RT&Tag uses native nuclei with intact Polycomb domains and nuclear speckles, making it ideal for studying nuclear compartments (Figure 1B, Figure S1A).

Using RT&Tag, we generated sequencing libraries from human female HEK293T cells targeting Polycomb domains with an antibody to the trimethylation of histone H3 at lysine-27 (H3K27me3), or targeting nuclear speckles with the SC35 antibody which recognizes the structural component of nuclear speckles, SRRM2^24^. We use a non-specific IgG antibody to generate a nucleoplasm control library. The RT&Tag dataset for each epitope showed high reproducibility between 3 biological replicates in HEK293T cells (r = 0.89-0.99, Figure S1B). Since Polycomb domains and nuclear speckles are nonoverlapping compartments^8^, we expected their RNA content to be distinct. Indeed, principal component analysis (PCA) showed that H3K27me3-, SC35-, and IgG-targeted RT&Tag libraries cluster into distinct groups (Figure 1C). Next, we used DESeq2^25^ to call transcripts differentially enriched in each compartment relative to IgG (>2-fold change and <0.05 Benjamini-Hochberg (BH) adjusted p-value). In total, transcripts from 3893 genes were enriched by H3K27me3-targeted RT&Tag, while transcripts from 1885 genes were enriched by SC35-targeted RT&Tag (Figure 1D, Tables S1). Transcripts from 351 genes (6.5%) are enriched in both datasets (Figure 1E).

We then asked whether RT&Tag recapitulates the enrichment of transcripts known to localize to each nuclear compartment. The *XIST* long noncoding RNA (lncRNA) is found on the H3K27me3-marked inactive X chromosome in mammalian female cells^4^, and *XIST* is strongly enriched in H3K27me3-targeted RT&Tag libraries (21-fold, Figure 1D, F). Given that reverse transcription in RT&Tag is primed with an oligo(dT), signal tends to be enriched near the 3’ end of this mature transcript. In contrast, the *MALAT1* lncRNA is a known constituent of nuclear speckles^26^, and is strongly enriched in SC35-targeted RT&Tag libraries (9-fold, Figure 1D, F). *MALAT1* is not-polyadenylated^11^, and thus reverse transcription is primed at A-rich stretches within the *MALAT1* transcript instead of at the 3’end (Figure 1F). As was seen using other methods^27,28^, we observe weak enrichment of *MALAT1* in H3K27me3-targeted RT&Tag libraries (3.4-fold, Figure 1E), potentially due to broad interactions of *MALAT1* with chromatin^29,30^. We obtained similar results for H3K27me3- and SC35-targeted RT&Tag in the K562 female cell line (Figure S1C-G, Tables S1). These results demonstrate that RT&Tag successfully detects transcripts enriched within nuclear compartments *in situ*.

### Long genes are transcribed adjacent to Polycomb domains.

**Figure 2:**
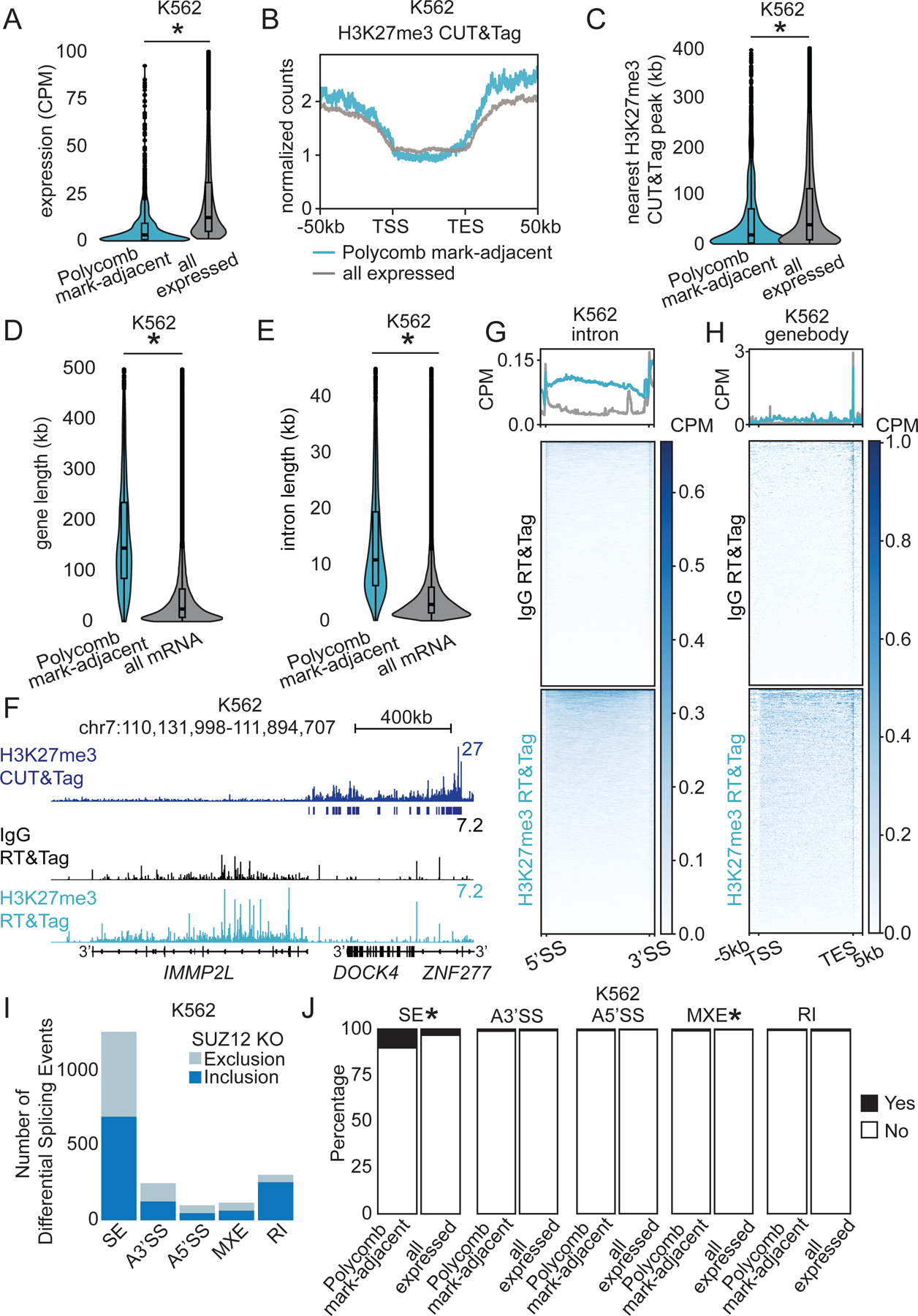
Long genes are transcribed adjacent to Polycomb domains. A) Violin plots showing RNA-seq expression of Polycomb mark-adjacent and all expressed transcripts in K562 cells. *p<0.05, unpaired t-test. CPM- counts per million. B) Profile plot of H3K27me3 CUT&Tag signal over gene bodies (±50kb) of Polycomb mark-adjacent and all expressed transcripts in K562 cells. C) Violin plots of distances from gene bodies of Polycomb mark-adjacent and all expressed transcripts to the nearest H3K27me3 CUT&Tag peak in K562 cells. *p<0.05, unpaired t-test. D-E) Violin plots of gene (D) and intron (E) lengths of Polycomb mark-adjacent and all annotated mRNA transcripts in K562 cells. *p<0.05, unpaired t-test. F) Genome track showing H3K27me3 CUT&Tag signal and peaks, along with IgG and H3K27me3-targeted RT&Tag signal over *IMMP2L* in K562 cells. G-H) Heatmaps of IgG and H3K27me3-targeted RT&Tag signal over introns (G) and gene bodies (H) of Polycomb mark-adjacent transcripts in K562 cells. I) Bar graph of differential splicing events (FDR<0.05) in SUZ12 knock-out K562 cells. J) Bar graph of percentage of Polycomb mark-adjacent or all expressed transcripts with differential splicing events in SUZ12 knock-out K562 cells. *p<0.05, chi-squared test. SE- skipped exon, A3’SS- alternative 3’splice site, A5’SS- alternative 5’splice site, MXE- mutually exclusive exons, RI- retained intron. See also Figure S2.

Our prior work in *Drosophila melanogaster* identified low levels of nascent transcription in Polycomb domains, including classic Polycomb-repressed *Hox* genes^21^. This implies that Polycomb-repressed genes may not always be completely silenced in Drosophila cells. In contrast, H3K27me3-targeted RT&Tag in K562 human cells does not recover *Hox* gene transcripts (Figure S2A). We do not believe this is due to low RT&Tag sensitivity as Polycomb-silenced *Hox* transcripts are also undetectable in a high read depth (~100 million paired reads) ENCODE K562 RNA sequencing dataset^31^. We then used published H3K27me3-targeted CUT&Tag data in K562 cells^32^ and compared it to our H3K27me3-targeted RT&Tag signal. We did not detect H3K27me3 RT&Tag signal enrichment over H3K27me3-marked chromatin (Figure S2B). However, the transcripts from 1390 genes recovered with H3K27me3-targeted RT&Tag in K562 cells are distinctive. They tend to be expressed at lower levels than all expressed genes (median 3.3 versus 20 CPM respectively, Figure 2A) even though these genes lack the H3K27me3 mark (Figure 2B). Instead, the H3K27me3 mark is present nearby (Figure 2B). The genes of the recovered transcripts are a median of 25kb from an H3K27me3-marked region, with only 19% directly overlapping a peak (Figure 2C & Figure S2C). Of those directly overlapping a peak, most had the H3K27me3 mark right at the borders of genes with little signal directly over gene bodies (Figure S2D). Thus, in human cells, H3K27me3-targeted RT&Tag detects transcripts from genes near Polycomb domains. We therefore will refer to these as Polycomb mark-adjacent transcripts.

Additional features of Polycomb mark-adjacent transcripts are distinctive. The genes producing these transcripts are typically very long (median length 156kb; Figure 2D). These long genes have long introns (median length 12.7kb; Figure 2E) and average length exons (Figure S2E). For example, transcripts from the 900kb *IMMP2L* gene (Figure 2F) were detected by H3K27me3-targeted RT&Tag. RT&Tag signal is enriched across the entirety of this gene, including its introns. In contrast, a Polycomb domain is upstream of *IMMP2L* across the *DOCK4* gene, but this gene is not expressed, and RT&Tag does not recover transcripts from it. The long gene and intron length features were not unique to K562 cells as Polycomb mark-adjacent transcripts identified in HEK293T cells showed a similar trend (Figure S2F) and genes found near H3K27me3 domains are in general long with long introns ( Figure S2G). Given that the transcripts detected by RT&Tag near Polycomb domains are exceptionally long, it is conceivable that they may contact nearby Polycomb domains during or after their transcription.

We were intrigued that H3K27me3-targeted RT&Tag captures signal throughout the gene body of *IMMP2L*. Oligo(dT) priming of reverse transcription captures both the 3’end of transcripts near the poly(A) tail and internal segments upstream of A-rich stretches. The signal mapping across the *IMMP2L* gene implies that intronic sequences are still present in the transcript when H3K27me3-targeted RT&Tag was performed. In fact, we observed extensive H3K27me3 RT&Tag signal over introns of most Polycomb mark-adjacent transcripts (Figure 2G). This implies that splicing has not yet occurred while Polycomb mark-adjacent transcripts are near chromatin.

Given these high levels of intronic signal, we wondered whether it comes from nascent Polycomb mark-adjacent transcripts. By definition, nascent transcripts are still engaged with RNA polymerase and so are not yet polyadenylated^33^. While we observed H3K27me3 RT&Tag signal at the 3’end of nearly all Polycomb mark-adjacent transcripts indicating priming of a poly(A) tail, it was no greater than that in IgG RT&Tag controls (Figure 2H). This finding suggests that these intronic signals are coming from nascent Polycomb mark-adjacent transcripts near Polycomb domains.

The localization of genes with exceptionally long introns adjacent to Polycomb domains is striking and may be beneficial to position them away from nuclear speckles, which contain a high concentration of splicing factors^8,34^. Thus, loss of Polycomb domains may result in mis-splicing of Polycomb mark-adjacent transcripts. Indeed, knock-out of SUZ12, the core component of the Polycomb repressive complex 2, in K562 cells^31^ results in over a thousand differential splicing events (FDR<0.05; Figure 2I). Of these, exon skipping events occur more frequently in Polycomb mark-adjacent transcripts than all expressed transcripts (10% vs 3%, Figure 2J), supporting our hypothesis that Polycomb domains may be important for the proper splicing of Polycomb mark-adjacent transcripts.

### SLAM-RT&Tag for metabolic labeling of RNA within nuclear compartments.

**Figure 3:**
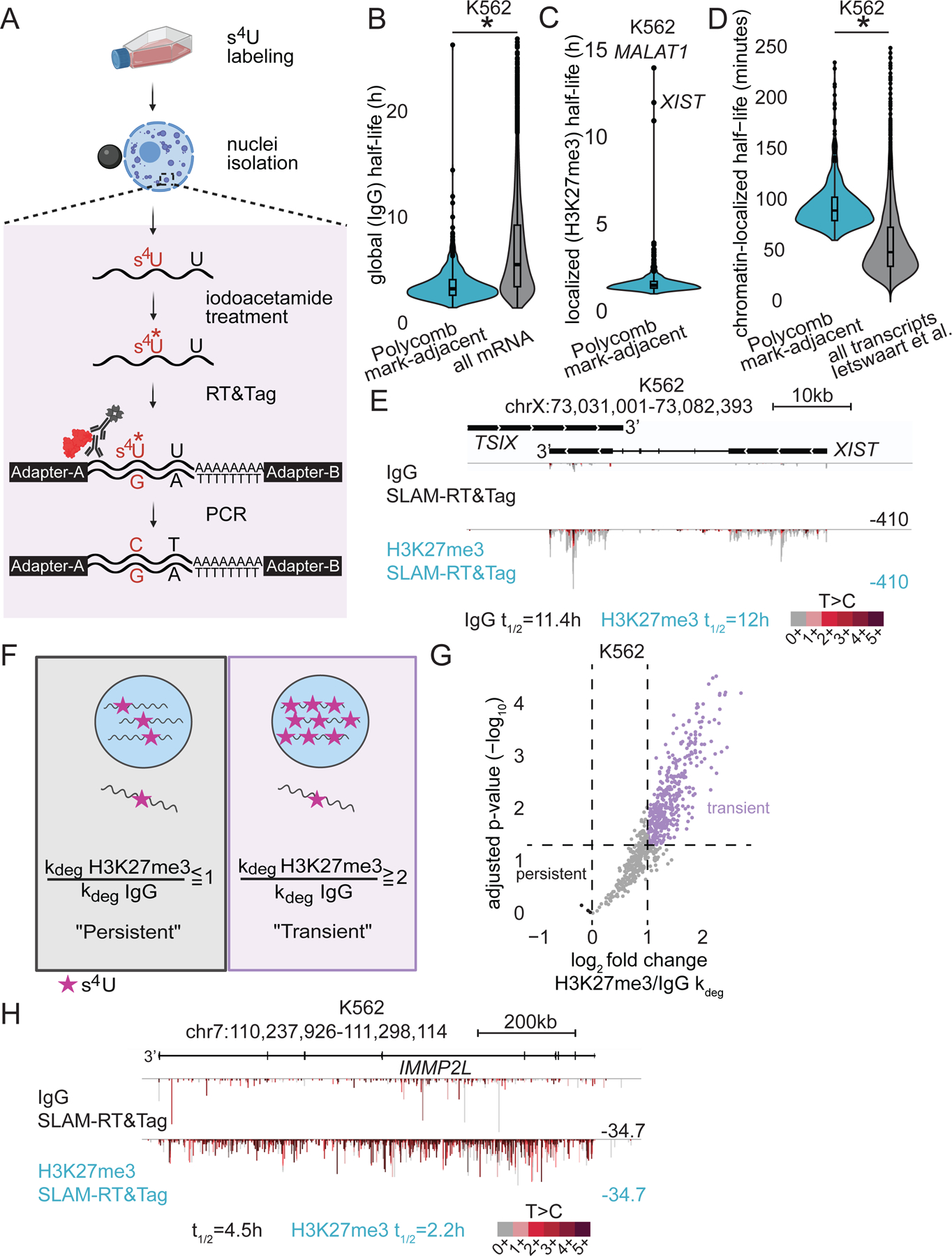
Polycomb mark-adjacent transcripts are transient near Polycomb domains. A) SLAM-RT&Tag schematic: 1) feeding cells with s^4^U; 2) nuclei isolation and bead binding; 3) iodoacetamide treatment to carboxyamidomethylate s^4^U; 4) standard RT&Tag steps; 5) introduction of T-C conversions during reverse transcription. B-C) Violin plots of global (IgG) (B) and localized (H3K27me3) (C) half-lives of Polycomb mark-adjacent and all expressed mRNA transcripts in K562 cells. *p<0.05, unpaired t-test. D) Violin plots of chromatin-localized half-lives of Polycomb mark-adjacent transcripts relative to all transcripts in the Ietswaart et al^41^ dataset in K562 cells. *p<0.05, unpaired t-test. E) Genome track showing IgG and H3K27me3-targeted SLAM-RT&Tag signal over *XIST* in K562 cells, half-lives (t_1/2_) listed below. F) Transcript classification: "Transient" (higher localized k_deg_ and s^4^U labeling than global) vs. "Persistent" (reverse trend). G) Volcano plots showing differential kinetic analysis of Polycomb mark-adjacent transcripts (k_deg_ log_2_ FC>2, FDR<0.05, n=3) in K562 cells. H) Genome track showing IgG and H3K27me3-targeted SLAM-RT&Tag signal over *IMMP2L* in K562 cells, halflives (t_1/2_) listed below. See also Figure S3.

Polycomb mark-adjacent transcripts have very long introns and gene bodies and so take a long time to be transcribed^35^. Splicing of long introns is challenging as the sites necessary for a splicing reaction are far apart and introns will undergo noncanonical recursive splicing reactions instead^36,37^. For these reasons, we were interested in measuring how long Polycomb mark-adjacent transcripts remain near chromatin. The thiol (SH)-linked alkylation for the metabolic sequencing of RNA (SLAM-seq) is a sequencing-based method that quantifies the percentage of transcripts metabolically labeled with a thiol-modified uridine analog, 4-thiouridine (s^4^U)^23^. We integrated steps of the SLAM-seq protocol into RT&Tag to develop SLAM-RT&Tag (Figure 3A). SLAM-RT&Tag begins by treating cells with 4-thiouridine (s^4^U) to metabolically label RNA, followed by nuclei isolation and binding to magnetic concanavalin A beads. Iodoacetamide (IAA) treatment is then performed to carboxyamidomethylate the s^4^U. The standard RT&Tag steps are then performed. During reverse transcription, the carboxyamidomethylated s^4^U is misread by the reverse transcriptase, resulting in a T>C conversion. Illumina sequencing libraries are then amplified, purified, and sequenced. Lastly, sequencing reads are aligned and the T>C conversion events are quantified using the SlamDunk software^38^.

To develop SLAM-RT&Tag, five critical points of the carboxyamidomethylation reaction were optimized. First, the IAA reaction was performed in 150 mM NaCl Wash buffer as the low salt SLAM-seq buffer (50 mM) caused nuclei clumping and RT&Tag failure (Figure S3A). Second, we lowered the IAA reaction temperature from 50°C to 37°C to prevent protein denaturation. Third, the reaction duration was increased from 15 minutes to 1 hour to compensate for the lower temperature. Fourth, the DMSO content of the IAA reaction was reduced from 50% to 10% of the total reaction volume. The 10% DMSO was sufficient to achieve high IAA reaction efficiency in the original SLAM-seq protocol^23^ and is compatible with RT&Tag which routinely uses nuclei frozen in 10% DMSO. Lastly, we found it imperative to perform the IAA reaction before antibody binding (Figure S3B). IAA can bind cysteine residues, thus IAA could destabilize disulfide bridge-rich proteins such as antibodies^39^. After implementing these five modifications, we generated SLAM-RT&Tag libraries that were comparable in yield to RT&Tag libraries (Figure S3C) and had sufficiently high levels of T>C conversion (Figure S3D)^38^.

We then confirmed that s^4^U labeling and IAA treatment did not disrupt the detection of transcripts within nuclear compartments. We found that a 4-hour s^4^U treatment resulted in sufficiently high rates of T>C conversion without affecting cell growth, gene expression, and transcript localization to compartments (Figures S3E-H). Likewise, samples generated with or without IAA treatment were highly correlated (r = 0.94-0.99) (Figure S3I) and did not affect compartment structure (Figure S3J). In summary, our data demonstrates that SLAM-RT&Tag is a suitable approach for metabolic labeling of RNA within nuclear compartments.

### Polycomb mark-adjacent transcripts are transient near Polycomb domains.

We applied SLAM-RT&Tag to examine the half-lives of Polycomb mark-adjacent transcripts. The BakR computation tool was used to estimate global half-lives using the IgG-targeted SLAM-RT&Tag dataset (nucleoplasmic control)^40^. BakR quantifies RNA degradation rates using a steady-state model of RNA dynamics. Specifically, BakR estimates the degradation rate constant (k_deg_) for transcripts corresponding to each gene using the T>C conversion rate and s^4^U pulse duration. Transcript half-lives (t_1/2_) are then computed using the t_1/2_=ln(2)/k_deg_ formula. Using BakR, we estimated the half-lives of transcripts corresponding to 48% of genes encoding Polycomb mark-adjacent transcripts that had sufficient reads for BakR data pre-processing. These transcripts have global half-lives with a median of 3.2 hours (Figure 3B).

We then calculated the localized half-lives of Polycomb mark-adjacent transcripts using the H3K27me3-targeted SLAM-RT&Tag dataset. The localized half-lives of Polycomb mark-adjacent transcripts were on average 1.6 hours, with 75% of half-lives being between 1 to 1.7 hours (Figure 3C). Half-lives of transcripts on chromatin were reported to range from 19 to 120 min in K562 cells (Figure 3D, Figure S3K)^41^. Thus, half-lives of Polycomb mark-adjacent transcripts on chromatin are longer than the average reported by subcellular TimeLapse-seq^41^, likely due to the longer time needed to finish transcribing long transcripts.

A few Polycomb mark-adjacent transcripts have exceptionally long localized half-lives (Figure 3C). One of them is *XIST*, a mature transcript retained on the Polycomb-marked X chromosome^4^. A transcript’s localized half-life encompasses the overall stability of the transcript and how quickly it leaves Polycomb domains. Since *XIST* is retained within Polycomb domains, its localized and global half-lives should be equal. Indeed, the localized half-life of *XIST* is nearly identical to its global half-life (12 vs 11.4 hours; Figure 3E). Hence, SLAM-RT&Tag recapitulates the retention of *XIST* at Polycomb domains.

We then asked whether other Polycomb mark-adjacent transcripts are retained near Polycomb domains. To address this question, we performed differential kinetic analysis using BakR. First, we estimated the global and Polycomb domain localized k_deg_ of Polycomb mark-adjacent transcript corresponding to each gene (Table S2). Next, we calculated the fold-change difference in localized and global k_deg_ constants and estimated Benjamini and Hochberg (BH) adjusted p-values. Transcripts from genes with a higher localized than global k_deg_ (H3K27me3/IgG k_deg_ ≥2 and adjusted p-value <0.05) were categorized as “transient” (Figure 3F). Alternatively, transcripts from genes with equal localized and global k_deg_ (H3K27me3/IgG k_deg_ ≤1) were classified as “persistent”. Over half of the genes producing Polycomb mark-adjacent transcripts are categorized as “transient” (Figure 3G). For example, a higher T>C conversion rate in H3K27me3- than IgG-targeted SLAM-RT&Tag is observed over the *IMMP2L* gene (Figure 3H). This finding supports Polycomb mark-adjacent transcripts being nascent transcripts that leave chromatin once processed. The other half of the genes producing Polycomb mark-adjacent transcripts are in-between “persistent” and “transient”. In contrast, only *XIST*, *MALAT1*, and *FANCL* are categorized as “persistent”. Hence, transcripts that persist at Polycomb domains, like *XIST*, are exceptions (Figure 3G). Instead, most transcripts picked up by H3K27me3-targeted RT&Tag are exceedingly long and are transcribed adjacent to Polycomb domains. Consistent with being nascent transcripts, Polycomb mark-adjacent transcripts are transient near Polycomb domains. Nevertheless, Polycomb mark-adjacent transcripts spend more time on chromatin than an average pre-mRNA, likely due to the longer time needed to transcribe their long gene bodies. The positioning of genes with exceptionally long introns near Polycomb domains is intriguing. Polycomb domains tend to localize in-between the nuclear speckles and the nuclear lamina^8,34^. Given the long time needed to transcribe their long introns, Polycomb mark-adjacent transcripts may benefit from being sequestered away from the high concentrations of RNA processing factors around nuclear speckles to prevent mis-splicing events.

### Nuclear speckles contain partially spliced polyadenylated transcripts.

**Figure 4:**
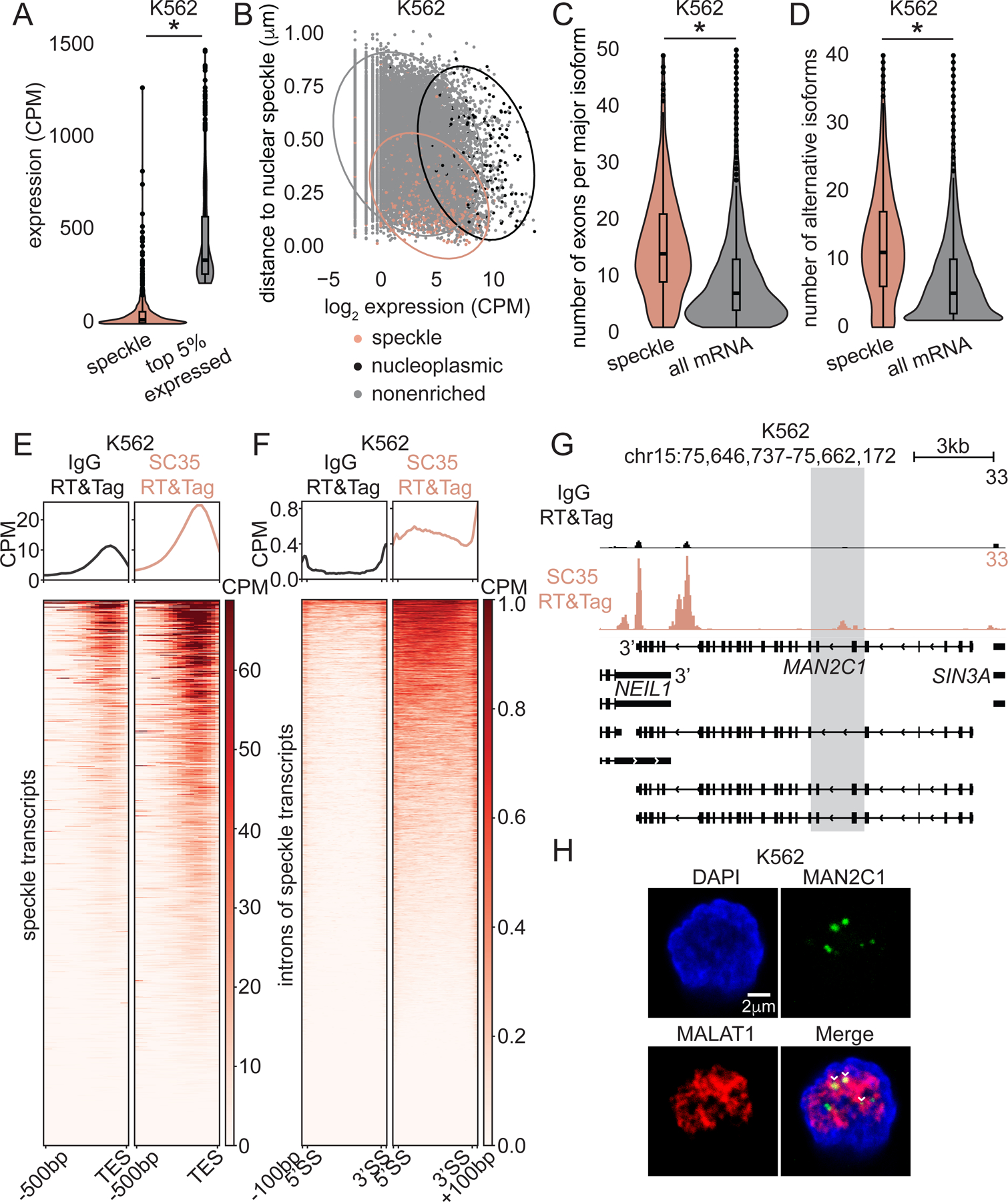
Nuclear speckles contain partially spliced polyadenylated transcripts. A) Violin plots of RNA-seq expression of speckle transcripts and top 5% expressed transcripts in K562 cells. *p<0.05, unpaired t-test. CPM-Counts per million. B) Scatter plot showing lack of correlation between gene expression (log_2_ CPM) and distance to nuclear speckle (μm, TSA-seq) in K562 cells. C-D) Violin plots of number of exons per major isoform (C) and alternative isoforms (D) of speckle transcripts and all annotated mRNA transcripts in K562 cells. *p<0.05, unpaired t-test. E and F) Heatmaps of IgG and SC35-targeted RT&Tag signal 500bp upstream of the 3’end (E) and within introns (F) of speckle transcripts in K562 cells. G) Genome track showing IgG and SC35-targeted RT&Tag signal over *MAN2C1* in K562 cells. H) RNA-FISH targeting *MALAT1* and *MAN2C1* intron (highlighted region in (F)) in K562 cells. Arrowheads mark overlap. Scale bars, 2μm. See also Figure S4.

Inspired by our findings with Polycomb mark-adjacent transcripts, we wondered whether the RNA processing mechanisms are different within a compartment enriched in splicing factors. Therefore, we focused on nuclear speckles, which are known to contain a high concentration of splicing factors as well as RNA^12,13,42^. We first defined speckle transcripts as transcripts enriched by SC35-targeted RT&Tag. Previous studies showed that highly transcriptionally active chromatin regions localize near nuclear speckles^8^. However, we did not observe high levels of active histone marks, H3K4me3 and H3K36me3, over the transcriptional start sites (TSS) or gene bodies of speckle transcripts, respectively (Figure S4A). Furthermore, speckle transcripts detected by RT&Tag were expressed at lower levels than the top 5% of expressed genes (Figure 4A) assessed by whole-cell RNA sequencing.

It is plausible that speckle transcripts are transcribed from distant genes and then migrate to nuclear speckles. We asked whether the proximity of a gene to a nuclear speckle and its expression is correlated. To do so, we turned to published Tyramide signal amplification sequencing (TSA-seq) data in K562 cells^8^. TSA-seq is a sequencing-based method that measures the proximity of chromosomal loci to nuclear speckles^8^. We determined the distance to nuclear speckles using TSA-seq and the expression level using RNA-seq for every expressed gene. The two features were only weakly negatively correlated (rho=-0.25) (Figure 4B). Likewise, weak negative correlation was observed between TSA-seq and ENCODE nascent RNA expression as measured using Bromouridine sequencing (Bru-seq)^31^ (rho=-0.22; Figure S4B). Thus, gene expression level plays at most a minor role in explaining proximity of genes encoding speckle transcripts to nuclear speckles.

To better understand why some transcripts localize to nuclear speckles, we explored the features of speckle transcripts. Intriguingly, speckle transcripts have twice the number of exons (Figure 4C) and isoform variants (Figure 4D) compared to the average mRNA. A similar trend is also present in speckle transcripts identified in HEK293T cells (Figure S4C). Having more exons implies that speckle transcripts have more complex splicing requirements. Localization to nuclear speckles may facilitate efficient splicing of speckle transcripts given the high concentration of splicing factors within nuclear speckles^22^.

RNA fluorescence *in situ* hybridization experiments have detected poly(A) signal within nuclear speckles^12,13,42^. Indeed, SC35-targeted RT&Tag signal is enriched upstream of the TES of speckle transcripts (Figure 4E), supporting the prior cytological observations. In addition to enrichment upstream of TES, SC35-targeted RT&Tag signal is enriched over ~30% of speckle transcript introns (Figure 4F). For example, the top speckle transcript hit *MAN2C1* is ~13kb in length and contains 26 exons (Figure 4G). SC35-targeted RT&Tag signal is enriched over both the 3’end of the gene and a few of its introns. RNA fluorescence *in situ* hybridization (RNA FISH) cytologically confirms the presence of the highlighted intron of *MAN2C1* within nuclear speckles (Figure 4H, Table S3). In addition to SC35, we performed RT&Tag targeting the structural components of nuclear speckles, SRRM2 and SON^24^, in HEK293T cells. SRRM2 and SON-targeted RT&Tag recapitulated most transcripts identified by SC35-targeted RT&Tag (Figure S4D) and captured signal enriched over ~30% of speckle transcript introns (Figure S4E). RT&Tag only captures signal from introns that contain A-rich stretches, hence the intronic content of speckle transcripts is likely underestimated. Nevertheless, our data illustrates that nuclear speckles contain polyadenylated transcripts that are incompletely spliced.

Nuclear speckle transcripts have been associated with retained introns (RI)^43^. RI is an alternative splicing event whereby an intron fails to be spliced out and is retained in mature mRNAs^44^. Hence, we asked whether speckle transcripts represent alternative splicing RI events or are in the process of being spliced. The lack of intronic signal within speckle transcripts in the nucleoplasm (Figure 4F) or whole-cell RNA-seq (Figure S4F&G) suggests the latter. This strongly supports the notion that nuclear speckles are sites of post-transcriptional splicing, consistent with active spliceosomes observed within nuclear speckles^18^.

### Incompletely spliced transcripts migrate to nuclear speckles.

**Figure 5:**
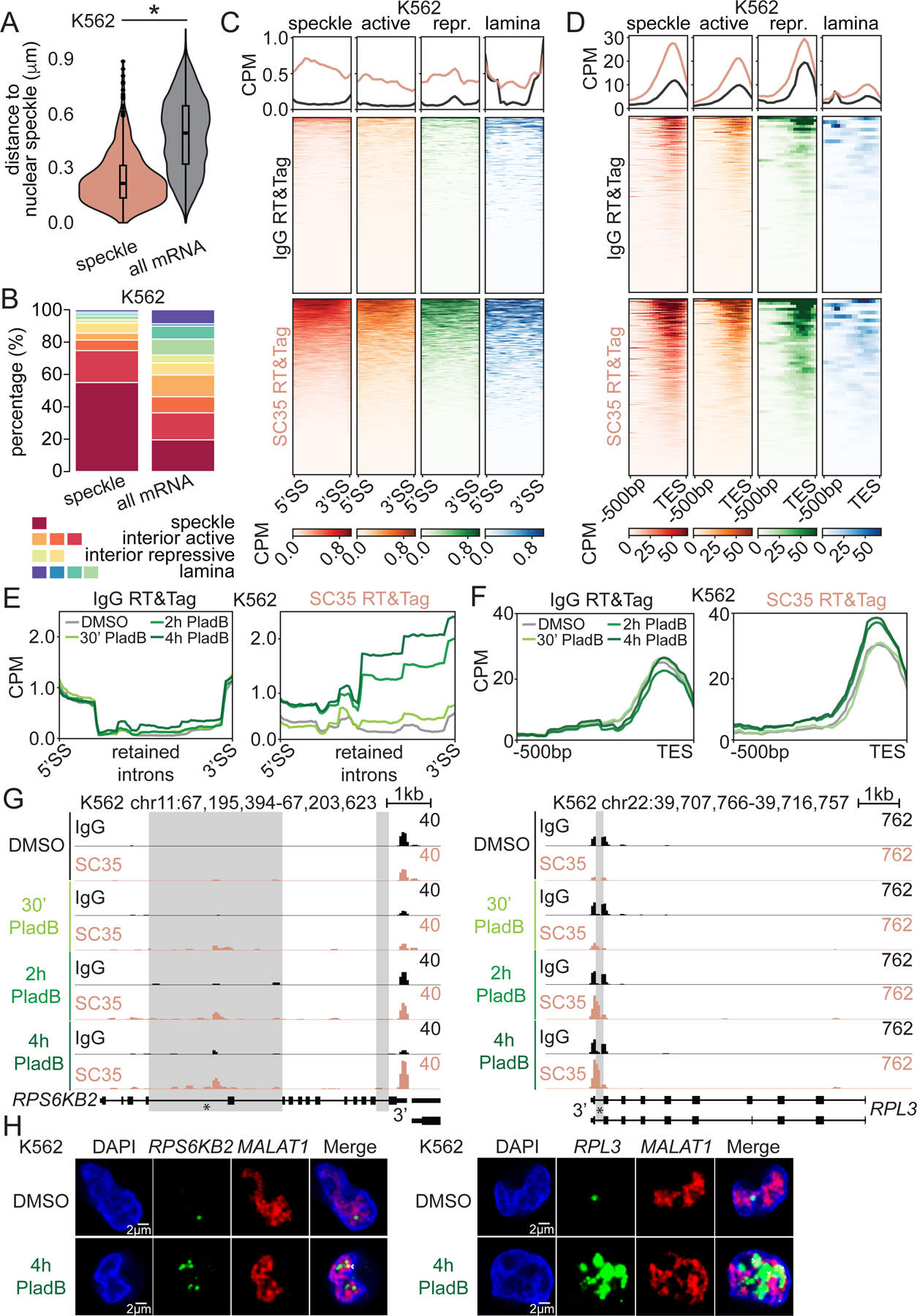
Incompletely spliced transcripts migrate to nuclear speckles. A) Violin plots of distances from gene bodies of speckle transcripts and all mRNA transcripts to nuclear speckles (TSA-seq) in K562 cells. *p<0.05, unpaired t-test. B) Stacked bar plots of percentage of speckle transcripts and all mRNA transcripts transcribed within SPIN states (“speckle”, “active”, “repressive”, and “lamina”) in K562 cells. C-D) Heatmaps of IgG and SC35-targeted RT&Tag signal over introns (C) and 500bp upstream of the 3’end (D) of speckle transcripts transcribed from different SPIN states in K562 cells. E-F) Profile plots of IgG and SC35-targeted RT&Tag over retained introns (E) and 500bp upstream of the 3’end of transcripts that gain retained introns (F) in PladB-treated K562 cells. G) Genome tracks showing IgG and SC35-targeted RT&Tag signal over *RPS6KB2* and *RPL3* in PladB-treated K562 cells. Retained introns are highlighted. H) RNA-FISH targeting *MALAT1* and introns (asterisk-marked regions in (F)) of *RPS6KB2* (left) and *RPL3* (right) in K562 cells treated with DMSO (top) or PladB (bottom) for 4h. Arrowheads mark overlap. Scale bars, 2μm. See also Figure S5.

Speckle transcripts are not transcribed directly within nuclear speckles, which lack chromatin^45^. Thus, we examined the proximity of speckle transcript transcription sites to nuclear speckles. Although genes producing speckle transcripts are closer to nuclear speckles than all genes producing expressed protein-coding transcripts, there is considerable variation in the distances (Figure 5A). To better define the transcriptional origins of speckle transcripts, we used published Spatial Position Inference of the Nuclear genome (SPIN) states data in K562 cells^34^. SPIN is a computational method that segments the genome into states which relate that segment’s positioning to nuclear compartments (nuclear speckle, nuclear lamina, and nucleolus)^34^. To assay, we intersected coordinates of speckle transcript-encoded genes with SPIN state genome segments. We found that only 55% of genes producing speckle transcripts are adjacent to nuclear speckles (Figure 5B, Table S4), thus the remaining transcripts must migrate to nuclear speckles.

If nuclear speckles are sites of post-transcriptional splicing, then polyadenylated transcripts with introns would migrate to them. Thus, we explored the features of speckle transcripts from different origins. To ensure enough transcripts in each transcriptional origin group, we consolidated SPIN states into 4 groups: speckle, active, repressive and lamina. Regardless of the transcriptional origin, ~30% of introns and nearly all TES of speckle transcripts are enriched for SC35-targeted RT&Tag signal (Figure 5C&D). As transcripts migrating to nuclear speckles are no longer chromatin-bound, we can infer they are most likely polyadenylated^46^. Hence, intronic signal enrichment should be from polyadenylated transcripts, substantiating that partially spliced polyadenylated transcripts from various transcriptional origins migrate to nuclear speckles.

Splicing inhibition results in retention of introns^47^ as well as enlarged nuclear speckles which accumulate poly(A)-containing RNA^18^. Therefore, we wondered whether incompletely spliced transcripts migrate to nuclear speckles in response to splicing inhibition. To test, we treated K562 cells with Pladienolide B (PladB), an inhibitor of the splicing factor SF3B1^48^. We then identified 458 significant RI events induced by 4 hours of PladB treatment (100 nM) using rMATS-turbo software^49^ (<0.05FDR, >3FC % intron inclusion) in a published RNA-sequencing dataset^47^ (Figure S5A, Table S5). PladB-induced retained introns have a large time-dependent gain in SC35-targeted RT&Tag signal (Figure 5E, Figure S5B), showing that these introns become enriched within nuclear speckles. Likewise, transcripts with PladB-induced RI events have an increase in SC35-targeted RT&Tag signal over their TES (Figure 5F, Figure S5C). The increase in SC35-targeted RT&Tag signal over the TES is modest, likely due to ~25% of genes encoding transcripts with PladB-induced RI events generating transcripts already being speckle transcripts (Figure S5D). The increase in SC35-targeted RT&Tag signal over introns that are not retained or become less retained and their corresponding transcripts is not observed (Figures S5E-H). Examples of transcripts with PladB-induced RI events migrating to nuclear speckles include the housekeeping genes, *RPS6KB2* and *RPL3*. Both have higher IgG than SC35-targeted RT&Tag signal under basal conditions (Figure 5G). Upon PladB treatment, *RPS6KB2* and *RPL3* gain SC35 RT&Tag signal over both TES and retained introns. Visualization of PladB-induced RI of *RPS6KB2* and *RPL3* by RNA-FISH show a similar trend (Figure 5H, Table S3). The RI of *RPS6KB2* and *RPL3* are found adjacent to nuclear speckles under basal conditions. Upon PladB treatment, the levels of these RI increase and begin to overlap with nuclear speckles. Therefore, our findings demonstrate that incompletely spliced transcripts, resulting from splicing inhibition, migrate to nuclear speckles.

### Speckle transcripts are predominantly transiently withheld in nuclear speckles.

**Figure 6.**
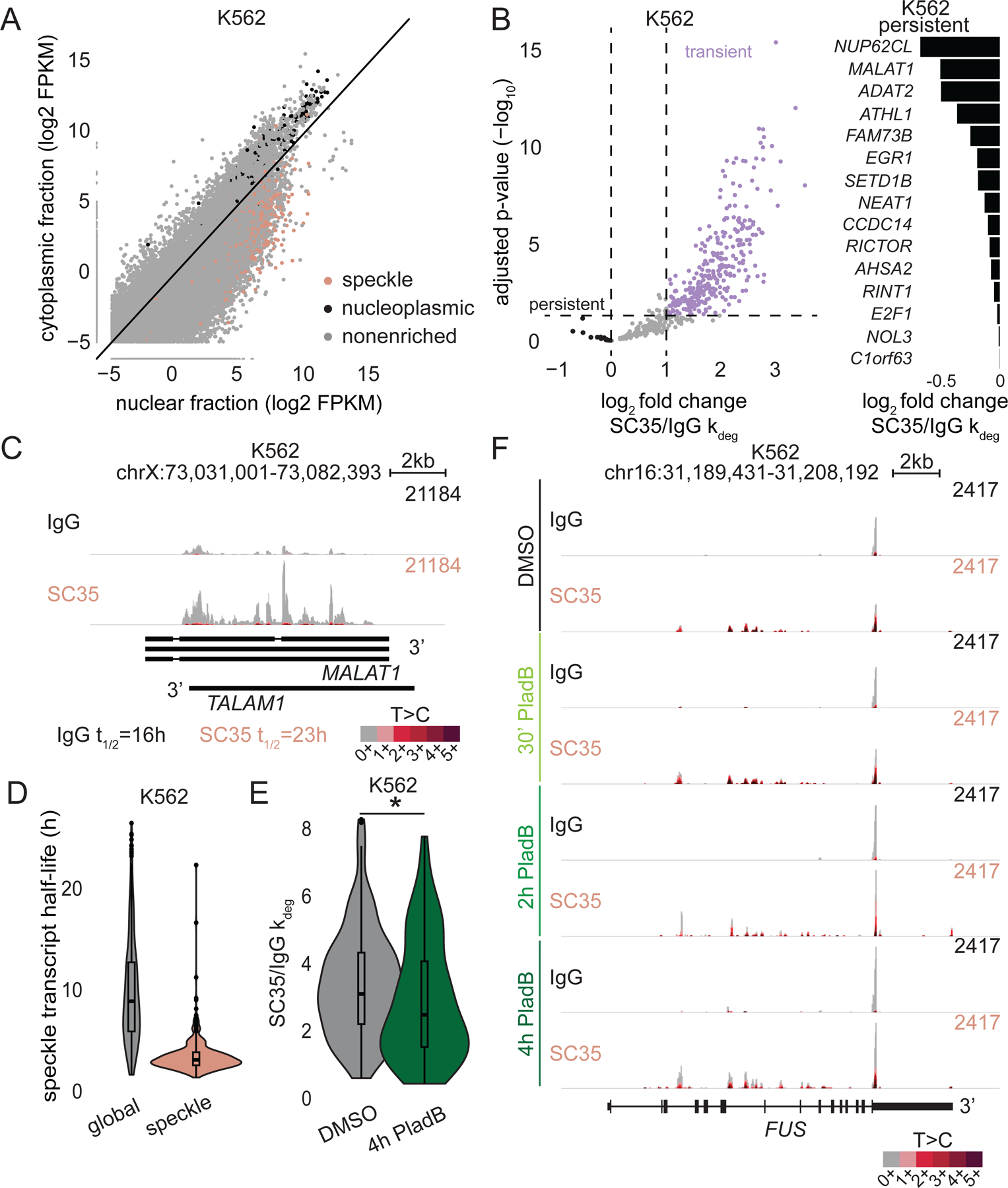
Speckle transcripts are predominantly transiently withheld in nuclear speckles. A) Correlation plot of transcript levels within nuclear and cytoplasmic fractions in K562 cells. FPKM-Fragments Per Kilobase of transcript per Million mapped reads. B) Volcano plot showing differential kinetic analysis of speckle transcripts (k_deg_ log_2_ FC>2, FDR<0.05, n=3) in K562 cells (left). Bar plot of SC35 over IgG k_deg_ for the top 15 persistent transcripts (right). C) Genome track showing IgG and SC35-targeted SLAM-RT&Tag signal over *MALAT1* in K562 cells, half-lives (t_1/2_) listed below. D) Violin plots of global (IgG) and localized (SC35) half-lives of speckle transcripts in K562 cells. *p<0.05, unpaired t-test. E) Violin plots of IgG over SC35 k_deg_ of speckle transcripts in K562 cells treated with DMSO or PladB for 4h. *p<0.05, unpaired t-test. F) Genome track showing IgG and SC35-targeted SLAM-RT&Tag signal over *FUS* in PladB-treated K562 cells, half-lives (t_1/2_) listed below. See also Figure S6.

Detained introns (DIs) are a subclass of retained introns present within mature transcripts that are detained in the nucleus ^50^. In response to stress stimuli, DI-containing transcripts complete splicing and subsequently are released into the cytoplasm^50^. It is plausible that speckle transcripts contain DIs and are withheld in the nuclear speckle until fully spliced. If so, one would expect amounts of speckle transcripts to be lower in the cytoplasm than in the nucleoplasm. We analyzed ENCODE RNA-seq data generated using RNA from K562 nuclear and cytoplasmic fractions. Indeed, nearly all speckle transcripts are low in the cytoplasmic fraction (Figure 6A), arguing that speckle transcripts are withheld in nuclear speckles.

If speckle transcripts require a stimulus to complete splicing and exit nuclear speckles, they should persist within nuclear speckles under basal conditions. To test this hypothesis, we turned to SLAM-RT&Tag and performed differential kinetic analysis using BakR to classify speckle transcripts as persistent or transient within nuclear speckles (Table S2). Only transcripts encoded by 15 genes are persistent within nuclear speckles (Figure 6B). One example is the lncRNA *MALAT1*, which exclusively localizes within nuclear speckles (Figure 6B&C)^26^. In contrast, 69% of speckle transcript-encoding genes generate transcripts that are transient within nuclear speckles (Figure 6B). This finding implies that transcripts are generally released from nuclear speckles without stress stimuli.

We then asked how long speckle transcripts reside within nuclear speckles. Using IgG and SC35-targeted SLAM-RT&Tag, we quantified global and nuclear speckle-localized halflives of speckle transcripts, respectively. Globally, speckle transcripts are stable, exhibiting halflives similar to those of average mRNA (Figure S6A). The localized half-lives of 75% of speckle transcripts ranged from 1.4 to 3.9 hours, with a median of 3.2 hours (Figure 6D). A localized half-life of 3.2 hours is quite long considering splicing takes in the order of minutes^51^. However, DIs are known to be spliced slowly (~1 hour)^50^, which may explain the long localized half-life if speckle transcripts contain DIs. We then explored features of transient versus persistent nuclear speckle transcripts. Persistent transcripts had fewer but longer introns than transient transcripts (Figure S6B). DIs tend to be longer than average introns^50^. If persistent transcripts harbor more DIs, they may be slower to splice and in-turn more persistent within nuclear speckles.

If release from nuclear speckles depends on splicing, speckle transcripts should persist for longer within nuclear speckles when splicing is inhibited. Indeed, speckle transcripts showed an overall decrease in SC35/IgG k_deg_ with 4-hour PladB treatment (Figure 6E, Table S6). For example, *FUS* is one of the most transient transcripts within the nuclear speckle. Upon PladB treatment, the T>C conversion decreased over the 3’end and introns of *FUS* (Figure 6F). PladB has the side effect of inhibiting transcriptional elongation, hence we performed flavopiridol treatment^47^. Comparable to flavopiridol, PladB treatment results in a lower overall T>C conversion rate (Figure S6C). However, flavopiridol treatment did not affect the persistence of speckle transcripts within nuclear speckles (Figure S6D). Hence, splicing inhibition increases transcript residency time within nuclear speckles independent of the effect on transcriptional elongation. Altogether, our results demonstrate that most transcripts are transient within nuclear speckles and their release depends on splicing. Nevertheless, some transcripts are persistent within nuclear speckles and may require a stress stimulus to be released.

### *SRSF11* transcripts are rapidly released from nuclear speckles in response to PladB treatment.

**Figure 7.**
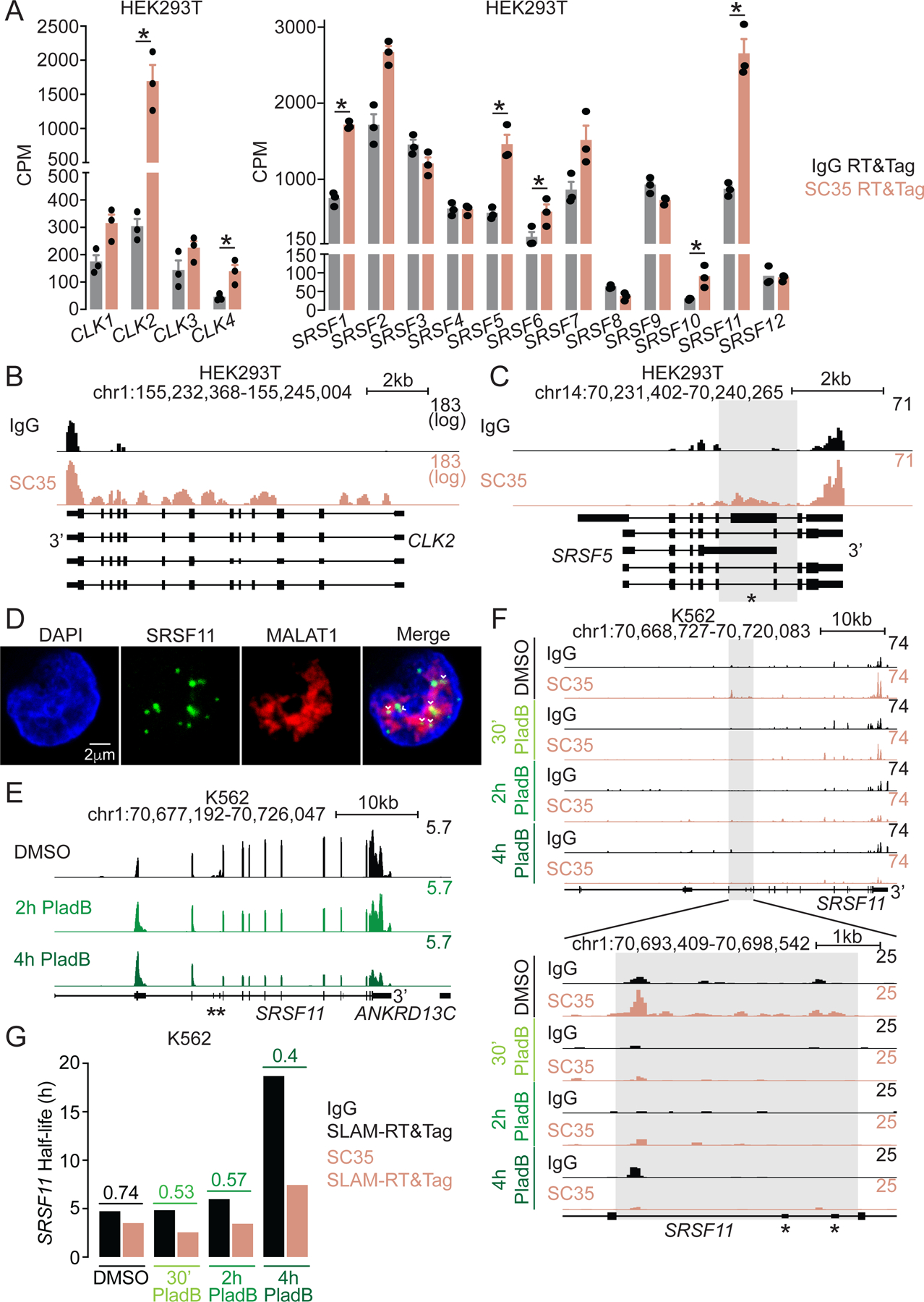
*SRSF11* transcripts are rapidly released from nuclear speckles in response to PladB treatment. A) Bar plot of IgG and SC35-targeted RT&Tag counts of *CLK* (left) and *SRSF* (right) transcripts in HEK293T cells. *p<0.05, BH-adjusted p-value. Data are represented as mean ± SEM. B-C) Genome track showing IgG and SC35-targeted RT&Tag signal over *CLK2* (B, log scale) and *SRSF5* (C) in HEK293T cells. The poison exon is marked with an asterisk and adjacent introns are highlighted. D) RNA-FISH targeting *MALAT1* and *SRSF11* intron (highlighted region in (F)) in K562 cells. Arrowheads mark overlap. Scale bar, 2μm. E-F) Genome track showing whole-cell RNA-sequencing (E) IgG and SC35-targeted RT&Tag signal (F) over *SRSF11* in PladB-treated K562 cells. Poison exons are marked with asterisks and adjacent introns are highlighted. G) Bar plot of global (IgG) and localized (SC35) half-lives of *SRSF11* in PladB-treated K562 cells. Fold change differences in localized/global half-lives are shown. See also Figure S7.

Splicing factor concentrations are auto-regulated via alternative splicing^52–54^. For example, Cdc2-like kinases (CLKs) have isoforms that lack a functional kinase domain^53^. Additionally, Serine/Arginine-rich splicing factors (SRSFs) have isoforms containing “poison exons” whose inclusion targets a protein for degradation^52^. Although included at low levels, even slight changes in poison exon inclusion lead to meaningful changes at the protein level^52^. Several *CLK* and *SRSF* transcripts are localized within nuclear speckles in K562 and HEK293T cells (Figure 7A, Figure S7A). In the case of *CLKs*, SC35-targeted RT&Tag signal is present over most of their introns (Figure 7B). In contrast, *SRSFs* within nuclear speckles have SC35-targeted RT&Tag signal over the introns surrounding poison exons (Figure 7C, Figure S7B). The introns surrounding the poison exons of *SRSF11* are also cytologically observed by RNA-FISH within nuclear speckles in K562 cells (Figure 7D, Table S3). It is plausible that incompletely spliced *SRSF* transcripts are withheld in nuclear speckles where their poison exons might be included or skipped in response to stimuli.

We looked at what happens to *SRSF* transcripts when splicing is globally inhibited with PladB. We specifically looked at *SRSF11*, which localizes within nuclear speckles in both K562 and HEK293T cells. In response to PladB treatment, overall *SRSF11* transcript levels are largely unchanged in K562 cells (Figure 7E, Figure S7C). There is however repositioning of RNA-seq signal towards the 5’end of *SRSF11*, consistent with PladB-induced transcriptional elongation impairment^47^. The two poison exons of *SRSF11* are spliced out upon PladB treatment (Figure 7F). We wondered whether *SRSF11* completes splicing within nuclear speckles. Interestingly, introns surrounding the two poison exons of *SRSF11* are rapidly spliced out by 30 minutes of PladB treatment (Figure 7F), likely independent from degradation by nonsense-mediated decay which occurs in the cytoplasm^52^. The levels of *SRSF11* transcript also gradually decline within nuclear speckles (Figure 7F, Figure S7D), suggesting it is released from nuclear speckles.

We then asked whether the rapid splicing of *SRSF11* results in its quicker release from nuclear speckles. Under basal conditions, *SRSF11* is not categorized as transient (1.35, SC35/IgG k_deg_) within nuclear speckles (Figure 7G). With PladB treatment, however, *SRSF11* becomes transiently associated (2.6, SC35/IgG k_deg_) with nuclear speckles, implying that it is released more quickly from nuclear speckles (Figure 7G). This observation is independent of reduced transcriptional elongation caused by PladB treatment (Figure S7E). Our findings demonstrate that nuclear speckles store incompletely spliced *SRSF11* transcripts. This process potentially provides a quick increase in SRSF11 protein levels in response to splicing inhibition and could have physiological implications as changes in SRSF11 levels have been associated with cancer^55,56^ and brain aging^57^.

## Discussion

Nuclear speckles contain polyadenylated RNA^12,13,42^, but the reasons for this remained speculative. Early RNA metabolic labeling and transcriptional inhibition experiments showed that nuclear speckles lack nascent transcripts^58,42^, suggesting nuclear speckles act as storage sites. Cytology and reporter construct-based studies later showed that transcripts transit through nuclear speckles to be spliced^15,16,59^ or to gain nuclear export competence^16,19,20^, challenging the storage site hypothesis. Hence, although multiple functions have been attributed to nuclear speckles, there is no consensus as to what exactly they do. Our work, along with a recent study^43^, showed speckles transcripts to have retained introns. We argue that these are not alternative splicing events but are incompletely spliced mature transcripts. We showed that incompletely spliced transcripts migrate to nuclear speckles in response to splicing inhibition, corroborating the idea that nuclear speckles are sites of post-transcriptional splicing^60^. Most transcripts are transient within nuclear speckles, arguing against nuclear speckles being long-term RNA storage sites. The relatively long half-lives of transcripts within speckles can explain earlier observations of them being poorly metabolically labeled. Additionally, we found that transcripts enriched within nuclear speckles are primarily retained in the nucleus and that their release from speckles is splicing-dependent. This finding implies that completion of splicing within nuclear speckles is necessary for nuclear export competence. Collectively, our observations reconcile the three dominant models of nuclear speckle function: storage, splicing, and nuclear export. We propose that nuclear speckles act as quality control checkpoints, temporarily storing incompletely spliced transcripts until splicing is completed post-transcriptionally, after which speckle transcripts can be exported to the cytoplasm. Such mechanism would ensure only fully spliced RNA molecules are exported to the cytoplasm.

Transcript dynamics offer insights into the biological processes occurring within nuclear speckles. Transcripts within nuclear speckles have a half-life of 2–3 hours. Likewise, RNA transcribed from a β-globin reporter was present within nuclear speckles for over 2 hours^16^. This long half-life is surprising as co-transcriptional splicing typically occurs within minutes^51^. Several factors may contribute to the long half-life of transcripts within nuclear speckles. First, transcripts need time to migrate to nuclear speckles, aging during this process. Second, some speckle transcripts may contain detained introns with weak splice acceptor and donor sites, delaying splicing^50^. Third, although splicing factors accumulate within nuclear speckles, they are not necessarily active. About 80–85% of active phosphorylated splicing factors are found within the chromatin fraction, while the remainder is within the nucleoplasm, where nuclear speckles are located^18^. Lastly, the TREX complex is implicated in the export of transcripts out of nuclear speckles^16^. Requiring an active process to export transcripts out of nuclear speckles may further increase their residency time.

Consistent with mammalian literature^61^, we did not detect transcription within Polycomb domains. Instead, we detect RNA transcribed from exceptionally long genes adjacent to Polycomb domains, likely due to their proximity to Polycomb domain- immunotethered pAG-Tn5 in 3D space. Long transcripts have been previously identified at the nuclear periphery, specifically the nuclear lamina^43,62^, albeit Polycomb domains are not exclusively found at the nuclear periphery^8,34^. Genes with long introns may have evolved to be adjacent to Polycomb domains to sequester them away from the high concentration of splicing factors near nuclear speckles^22,63^. This may avoid premature internal splicing before the long introns are fully transcribed. In contrast, mature transcripts containing introns that failed to be spliced co-transcriptionally migrate to nuclear speckles, where the high abundance of splicing factors likely aids in their post-transcriptional splicing. Thus, nuclear compartments contribute to RNA processing by exposing transcripts to specific splicing factors concentrations.

### Limitations of the study

RT&Tag is an *in situ*-based method that captures signals from transcripts near an epitope of interest. Consequently, the assignment of transcripts to nuclear compartments is limited by the proximity of immunoreagents to transcripts within the nucleus. Future assays with more precise labeling radii will help refine subnuclear positions.

## Resource availability

### Lead Contact

Further information and requests for resources and reagents should be directed to and will be fulfilled by the lead contact, Steven Henikoff (steveh@fredhutch.org).

### Materials Availability

All unique/stable reagents generated in this study are available from the lead contact with a completed materials transfer agreement.

### Data and Code Availability

All primary sequencing data have been deposited as single-end fastq files in the Gene Expression Omnibus under accession code GSE272219. Custom code for analyzing RT&Tag and SLAM-RT&Tag datasets is available at https://github.com/nadiyakhyzha/SLAMRTTag.

## STAR Methods

### Experimental model and study participant details

#### Cell Lines

HEK293T cells (female) were cultured in DMEM with GlutaMAX media (Gibco 10569010) supplemented with 10% FBS (Cytiva SH30070.03) and 1x Antibiotic-Antimycotic (Gibco 15240062) at 37°C and 5% CO_2_. K562 cells (female) were cultured in IMDM media (ATCC 30-2005) supplemented with 10% FBS (Cytiva SH30070.03) at 37°C and 5% CO_2_. Both HEK293T and K562 cells were maintained at sub-confluency and were passaged every 2–3 days. Cell numbers and cell viability were quantified using the Vi-CELL BLU cell viability analyzer (Beckman Coulter).

### Method details

#### Antibodies and drug treatments

The following primary antibodies were used for RT&Tag and SLAM-RT&Tag: rabbit anti-IgG (Abcam ab172730), rabbit anti-H3K27me3 (Cell Signaling Technology CST9733), mouse anti-SC35 (Abcam ab11826), rabbit anti-SON (Novus Biologicals NBP1–88706) and rabbit anti-SRRM2 (Thermo Scientific PA5–66827). The following secondary antibodies were used for RT&Tag and SLAM-RT&Tag: Guinea Pig anti-Rabbit (Antibodies Online ABIN101961) and Rabbit anti-Mouse (Abcam ab46450). Streptavidin-conjugated secondary antibodies were prepared using the Streptavidin Conjugation Kit (Abcam ab102921) as per the manufacturer’s instructions. All antibodies were used at a 1:100 dilution.

For immunofluorescence experiments the following primary antibodies were used: rabbit anti-H3K27me3 (Cell Signaling Technology CST9733, 1:250) and mouse anti-SC35 (Abcam ab11826, 1:200). The following secondary antibodies were used: 488 goat anti-rabbit (Thermo Fisher Scientific A11008, 1:500) or Cy5 goat anti-mouse (Jackson ImmunoResearch 115-175-166, 1:200).

For drug treatments, media was supplemented with 100 nM Pladienolide B (Cayman Chemical Company 16538), 1 μM Flavopiridol (Sigma-Aldrich F3055) or dimethylsulfoxide.

### RT&Tag

RT&Tag was performed using fresh cells as described previously^21^. A detailed step-by-step protocol can be found at: (https://www.protocols.io/view/rt-amp-tag-reverse-transcribe-amp-tagment-x54v9jyjqg3e/v1 ). Briefly, nuclei were isolated from 4 million mammalian cells by resuspending cell pellets in NE1 buffer (10 mM HEPES pH 7.9, 10 mM KCl, 0.1% Triton X-100, 20% glycerol, 0.5 mM spermidine, Roche Complete Protease Inhibitor Cocktail, 1 U/μL RNasin Ribonuclease Inhibitor) and incubating on ice for 10 minutes. The nuclei pellets were then collected and resuspended in Wash Buffer (20 mM HEPES pH 7.5, 150 mM NaCl, 0.5 mM spermidine, Roche Complete Protease Inhibitor Cocktail, 1 U/μL RNasin Ribonuclease Inhibitor). For each reaction, 200,000 mammalian nuclei were bound to 5 μL of Concanavalin A (ConA) beads (Bangs Laboratories BP531). The ConA-bound nuclei were incubated with primary antibody (1:100 in Antibody Buffer: 20 mM HEPES pH 7.5, 150 mM NaCl, 0.5 mM spermidine, Roche Complete Protease Inhibitor Cocktail, 2 mM EDTA, 0.1% BSA, and 1 U/μL RNasin Ribonuclease Inhibitor) overnight at 4°C. Subsequently, the nuclei were incubated with streptavidin-conjugated secondary antibody (1:100 in Wash Buffer) for 45 minutes at room temperature. After two washes with Wash Buffer, nuclei were incubated with biotinylated oligo(dT)-ME-B (0.2 mM in Wash Buffer) for 20 minutes at room temperature, followed by two more washes. Finally, nuclei were incubated with ME-A loaded pAG-Tn5 (Epicypher 15–1025; 1:200 in 300 Wash Buffer: 20 mM HEPES pH 7.5, 300 mM NaCl, 0.5 mM spermidine, Roche Complete Protease Inhibitor Cocktail, and 1 U/μL RNasin Ribonuclease Inhibitor) for 1 hour at room temperature and then washed twice with 300 Wash Buffer. Simultaneous reverse transcription and tagmentation were performed by resuspending nuclei in Reverse Transcription master mix (1x Maxima RT Buffer, 0.5 mM dNTPs, 10 U/μL Maxima H Minus Reverse Transcriptase, and 1 U/μL RNasin Ribonuclease Inhibitor; EP0752) and incubating for 2 hours at 37°C. The nuclei were then washed with 10 mM TAPS, and pAG-Tn5 was stripped off by resuspending the nuclei in 5 μL of Stripping Buffer (10 mM TAPS with 0.1% SDS) and incubating for 1 hour at 58°C. The SDS was quenched by adding 15 μL of 0.67% Triton X-100 to each tube. Libraries were then generated via PCR using the NEBNext High-Fidelity PCR Master Mix (NEB M0541L). The sequencing libraries were purified using 0.8x HighPrep PCR Cleanup System (MagBio AC-60500) beads. Library concentrations were quantified with the High Sensitivity D5000 TapeStation system (Agilent 5067–5592).

#### SLAM-RT&Tag

For RNA metabolic labeling, 100 μM s^4^U (Sigma-Aldrich T4509) was added to the cell media for 4 hours. In the case of SLAM-RT&Tag, K562 cells were pre-treated with s^4^U for 15 minutes before commencing 4-hour Pladienolide B treatment. After s^4^U treatment, cells were collected by centrifuging at 300*g* for 5 min followed by a wash with 1× PBS. Nuclei pellets were then resuspended in NE1 buffer (10 mM HEPES pH 7.9, 10 mM KCl, 0.1% Triton X-100, 20% glycerol, 0.5 mM spermidine, Roche Complete Protease Inhibitor Cocktail, 1 U/μL RNasin Ribonuclease Inhibitor) and left incubating on ice for 10 minutes. The nuclei were then centrifuged at 500g for 8 min and resuspended in Wash Buffer (20 mM HEPES pH 7.5, 150 mM NaCl, 0.5 mM spermidine, Roche Complete Protease Inhibitor Cocktail, 1 U/μL RNasin Ribonuclease Inhibitor). Per reaction, 200,000 mammalian nuclei were bound to 5 μL of Concanavalin A (ConA) beads (Bangs Laboratories BP531) for 10 minutes. The Wash Buffer was then removed and iodoacetamide reaction was performed by resuspending nuclei with iodoacetamide (10 mM, Sigma-Aldrich I1149, in Wash Buffer) and incubating for 1 hour at 37°C. The nuclei were then washed twice with Wash Buffer. The rest of the protocol was identical to RT&Tag, starting with overnight incubation with primary antibodies.

#### RNA-sequencing

Total RNA was isolated from K562 cells using the RNeasy Plus Mini Kit (Qiagen). First-strand synthesis was carried out with Maxima H Minus Reverse Transcriptase (Thermo Fisher Scientific EP0752), using the oligo(dT)-ME-B fusion oligonucleotide for priming. Tagmentation was then performed with 100 ng of RNA-cDNA hybrids, ME-A loaded pAG-Tn5 (Epicypher), and tagmentation buffer (20 mM HEPES pH 7.5, 150 mM NaCl, 10 mM MgCl2) for 1 hour at 37°C. The tagmented RNA-cDNA hybrids were purified using the HighPrep PCR Cleanup System (MagBio AC-60500) at a 1x ratio. Sequencing libraries were then amplified with 12 cycles of PCR using the NEBNext Master Mix (NEB M0541L) and purified with a 0.8x ratio of HighPrep PCR Cleanup System (MagBio AC-60500). Finally, libraries were quantified using the High Sensitivity D5000 TapeStation system (Agilent 5067–5592).

#### Immunofluorescence

To start, 12-well glass bottom plates (Fisher Scientific NC0799106) or 8-well glass chamber slides (Millipore PEZGS0816) were freshly coated with 0.01% poly-L-lysine (Sigma P4707) for 1 hour, followed by air drying for 1 hour. HEK293T or K562 cells were collected by centrifugation (300g for 5 min) and washed once with 1x PBS. Nuclei were isolated by resuspending cell pellets in NE1 buffer (10 mM HEPES pH 7.9, 10 mM KCl, 0.1% Triton X-100, 20% glycerol, 0.5 mM spermidine, Roche Complete Protease Inhibitor Cocktail, 1 U/μL RNasin Ribonuclease Inhibitor) and incubating on ice for 10 minutes. Nuclei were pelleted by centrifugation (500g for 8 min) and resuspended in 1x PBS. For iodoacetamide treatment, nuclei were resuspended in Wash buffer supplemented with 10% DMSO and 10mM iodoacetamide and incubated for 1 hour at 37°C. Approximately 650,000 nuclei were seeded per well onto the poly-L-lysine-coated 12-well plate and allowed to bind for 15 minutes. Either 400,000 cells or nuclei were seeded per well of 8-well glass chamber slides. Next, 16% formaldehyde was added directly to each well to achieve a final concentration of 4%. Nuclei were fixed for 10 minutes, followed by removal of formaldehyde and three rounds of PBS washes. Nuclei were permeabilized in 0.1% Triton X-100 in PBS for 10 minutes, then blocked in 1% BSA in PBS for 3 hours. Primary antibodies were added in 1% BSA in PBS and left overnight at 4°C. The next day, primary antibodies were removed, and nuclei underwent three rounds of PBS washes. Secondary antibodies were added in 1% BSA in PBS and incubated for 1 hour at room temperature. After incubation, secondary antibodies were removed, and nuclei were washed three times with PBS. Nuclei were stained with 0.1 µg/ml DAPI (Sigma D9542) in PBS for 10 minutes, followed by three rounds of PBS washes and a final wash with water. Mounting was performed using 10% glycerol. Images were acquired using a Leica SP8 confocal, with subsequent image processing using Fiji software.

#### RNA fluorescence *in situ* hybridization (RNA-FISH)

The RNAscope™ Multiplex Fluorescent Reagent Kit v2 (Advanced Cell Diagnostics 323270) was used for RNA-FISH experiments following the manufacturer’s instructions. Briefly, 8-well glass chamber slides (Millipore PEZGS0816) were freshly coated with 0.01% poly-L-lysine (Sigma P4707) for 1 hour, then air dried for 1 hour. K562 cells were collected by centrifugation (300g for 5 min) and washed once with 1x PBS. Approximately 400,000 K562 cells were seeded per well of 8-well glass chamber slides and allowed to adhere at room temperature for 15 minutes. Cells were fixed for 30 minutes in 10% Neutral-buffered formalin (Sigma-Aldrich HT501128) at room temperature. Alcohol dehydration was performed using a 50%/70%/100% ethanol gradient, followed by rehydration in a 70%/50% ethanol and PBS gradient. Hydrogen peroxide treatment was performed for 10 minutes at room temperature, followed by digestion with Protease III (1:20 dilution in PBS) for 10 minutes. Subsequently, RNAscope probes targeting MALAT1 (Advanced Cell Diagnostics 578171-C3, 1:150 dilution) or retained intron sequences of MAN2C1, SRSF11, RPL3, RPS6KB2 (Advanced Cell Diagnostics custom, undiluted) were hybridized for 2 hours at 40°C. Amplification and fluorophore conjugation steps were performed as per the manufacturer’s instructions. The following fluorophores were used: TSA Vivid Fluorophore 520 (1:1500, Advanced Cell Diagnostics 323271) and TSA Vivid Fluorophore 650 (1:3000, Advanced Cell Diagnostics 323271). Slides were mounted in ProLong® Diamond Antifade Mountant (Thermo Fisher Scientific P36965) and cured overnight at room temperature. Images were acquired using the Leica SP8 confocal and were processed using Fiji software^64^. Quantification of the RNA-FISH signal overlap between *MALAT1* and retained introns of nuclear speckle transcripts was performed using Fiji software (Table S3).

### Quantification and Statistical Analysis

#### Sequencing and data preprocessing

For RT&Tag, SLAM-RT&Tag, and RNA-sequencing, single-end 100 base pair sequencing was performed on the Illumina NextSeq 2000. RT&Tag sequencing reads are stranded (forward strand) and the strand information is considered during data pre-processing. RT&Tag and RNA-sequencing reads were aligned using HISAT2 to the UCSC hg19 genome with the options: -- max-intronlen 30000 --rna-strandness F^65^ (Table S7). The aligned reads were then quantified using featureCounts with the GENCODE GRCh37.p13 v19 gene annotation file using the following options: 1) -s 1 -t exon -g gene_id ; or 2) -s 1 -t transcript -gene_id ^66^. The second option was used for identifying Polycomb mark-adjacent transcripts by counting reads aligning to introns. Differential expression and principal component analysis were performed using DESeq2^25^.

SLAM-RT&Tag sequencing reads were aligned using the SLAM-DUNK^38^ pipeline to the UCSC hg19 genome using default options (Table S7). The SLAM-DUNK output BAM files were then sorted with SAMtools^67^ using the following options: samtools sort -n -o. The sorted BAM files were inputted into the bam2bakR pipeline^68^ using the default options to generate tdf files for genome browser visualization and cB files for subsequent analysis. The cB file was then used to estimate k_deg_ constants and perform differential kinetic analysis using BakR^40^. Half-lives (t_1/2_) were calculated using the t_1/2_=ln(2)/k_deg_ formula. For comparison with subcellular TimeLapse-seq, chromatin half-lives were retrieved from Ietswaart et al^41^.

For CUT&Tag analysis, published data was used^32^ and analyzed as described prior (dx.doi.org/10.17504/protocols.io.bjk2kkye)^69^. H3K27me3 peaks were called using SEACR with the following options: 0.01 non stringent^70^.

Profile plots, heatmaps, correlation matrices and bigWig files were generated using deepTools^71^. Distance to nuclear speckles was calculated using published TSA-seq^8^ with the multiBigwigSummary tool from deepTools^71^. SPIN^34^ states were assigned using the SPIN state genome segment and speckle transcript-encoding gene coordinates utilizing the findOverlaps function of GenomicRanges tool^72^ in Bioconductor. Retained intron events were identified using published RNA-sequencing data^47^ and rMATS-turbo^49^ software. Read count tables for RNA-sequencing data on nuclear (ENCSR530NHO) and cytoplasmic (ENCSR384ZXD) fractions in K562 cells were retrieved from the Encyclopedia of DNA Elements (ENCODE) project^31^. Read count tables for Bru-sequencing data (ENCSR729WFH) in K562 cells were retrieved from the Encyclopedia of DNA Elements (ENCODE) project^31^. RNA-sequencing data from K562 cells with CRISPR/Cas9 SUZ12 deletion were retrieved from the ENCODE project^31^ (ENCSR682DZY) and differential splicing events were identified using rMATS-turbo^49^ software. Genome browser tracks were visualized with Integrative Genomics Viewer (IGV)^73^ using bigWig or tdf files. Graphs were plotted using R Studio (https://www.r-project.org) using base graphics or using ggplot2 (https://ggplot2.tidyverse.org).

## Supplementary Material

1

2

3

4

5

6

7

8

## Figures and Tables

**Table:** Key resources table

REAGENT or RESOURCE	SOURCE	IDENTIFIER
Antibodies		
rabbit anti-IgG	Abcam	Cat# ab172730; RRID:AB_2687931
rabbit anti-H3K27me3	Cell Signaling Technology	Cat# CST9733; RRID: AB_2616029
mouse anti-SC35	Abcam	Cat# ab11826; RRID:AB_298608
rabbit anti-SON	Novus Biologicals	Cat# NBP1–88706; RRID:AB_11006030
rabbit anti-SRRM2	Thermo Scientific	Cat# PA5–66827; RRID:AB_2665182
guinea pig anti-rabbit	Antibodies Online	Cat# ABIN101961; RRID: AB_10775589
rabbit anti-mouse	Abcam	Cat# ab46540; RRID: AB_2614925
488 goat anti-rabbit	Thermo Fisher Scientific	Cat# A11008; RRID:AB_143165
Cy5 goat anti-mouse	Jackson ImmunoResearch	Cat# 115–175-166; RRID:AB_2338714
Chemicals, peptides, and recombinant proteins		
Pladienolide B	Cayman Chemical Company	Cat# 16538
Flavopiridol	Sigma-Aldrich	Cat# F3055
pAG-Tn5	Epicypher	Cat# 15–1025
s^4^U	Sigma-Aldrich	Cat# T4509
Iodoacetamide	Sigma-Aldrich	Cat# I1149
Roche Complete mini EDTA free protease inhibitor cocktail	Sigma-Aldrich	Cat# 11836170001
Rnasin Rnase Inhibitor	Promega	Cat# N2515
Concanavalin A paramagnetic beads	Bangs Laboratories	Cat# BP531
DAPI	Sigma	Cat# D9542
ProLong® Diamond Antifade Mountant	Thermo Fisher Scientific	Cat# P36965
TSA Vivid Fluorophore 520	Advanced Cell Diagnostics	Cat# 323271
TSA Vivid Fluorophore 650	Advanced Cell Diagnostics	Cat# 323271
Critical commercial assays		
Streptavidin Conjugation Kit	Abcam	Cat# ab102921
NEBNext High-Fidelity PCR Master Mix	NEB	Cat# M0541L
Maxima H Minus Reverse Transcriptase	Thermo Fisher Scientific	Cat# EP0752
RNAscope™ Multiplex Fluorescent Reagent Kit v2	Advanced Cell Diagnostics	Cat# 323270
HighPrep PCR Cleanup System	MagBio	Cat# AC-60500
High Sensitivity D5000 TapeStation system	Agilent	Cat# 5067–5592
Deposited data		
RT&Tag of nuclear compartments	This study	GSE272219
SLAM-RT&Tag of nuclear compartments	This study	GSE272219
K562 RNA-seq	This study	GSE272219
TSA-seq in K562 cells	Chen et al.^8^	GSE66019
SPIN states in K562 cells	Wang et al.^34^	GSE148362
subcellular TimeLapse-seq in K562 cells	Ietswaart et al.^41^	GSE207924
Retained introns in PladB treated K562 cells	Castillo-Guzman et al.^47^	GSE148768
Bru-sequencing data in K562 cells	ENCODE	ENCSR729WFH
RNA-seq in nuclear and cytoplasmic fractions in K562 cells	ENCODE	ENCSR530NHO, ENCSR384ZXD
RNA-seq in SUZ12 knock-out K562 cells	ENCODE	ENCSR682DZY
H3K27me3, H3K4me3 and K3K36me3 CUT&Tag in K562 cells	Henikoff et al.^32^	GSE158327
Experimental models: Cell lines		
K562	ATCC	Cat# CCL-243; RRID: CVCL_0004
HEK293T	ATCC	Cat# CRL-3216; RRID:CVCL_0063
Oligonucleotides		
RNAscope™ Probe-Hs-MALAT1-O7-C3	Advanced Cell Diagnostics	Cat# 578171-C3
RNAscope™ Probe - Hs-MAN2C1-O1-C1	Advanced Cell Diagnostics	Cat# 1731751-C1
RNAscope™ Probe - Hs-RPS6KB2-O2-C1	Advanced Cell Diagnostics	Cat# 1740701-C1
RNAscope™ Probe - Hs-RPL3-O2-C1	Advanced Cell Diagnostics	Cat# 1740691-C1
RNAscope™ Probe - Hs-SRSF11-O3-C1	Advanced Cell Diagnostics	Cat# 1639221-C1
Biotinylated-Oligod(T)-MEB: /5Biosg/GTCTCGTGGGCTCGGAGATGTGTATAAGAGACAGTTTTTTTTTTTTTTTTTTTTTTTTTTTTTTTVN	IDT	NA
Mosaic end_reverse: [PHO]CTGTCTCTTATACACATCT	IDT	NA
Mosaic end_Adapter A: TCGTCGGCAGCGTCAGATGTGTATAAGAGACAG	IDT	NA
Software and algorithms		
HISAT2	Kim et al.^65^	http://daehwankimlab.github.io/hisat2/
featureCounts	Liao et al.^66^	https://subread.sourceforge.net/
DESeq2	Love et al.^25^	https://github.com/mikelove/DESeq2
SAMtools	Li et al.^67^	http://www.htslib.org/
SLAM-DUNK	Neumann et al.^38^	https://t-neumann.github.io/slamdunk/
bam2bakR	Schofield et al.^68^	https://github.com/simonlabcode/bam2bakR
bakR	Vock et al.^40^	https://github.com/simonlabcode/bakR
SEACR	Meers et al.^70^	https://github.com/FredHutch/SEACR
deepTools	Ramirez et al.^71^	https://deeptools.readthedocs.io/en/develop/
rMATS-turbo	Wang et al.^49^	https://github.com/Xinglab/rmats-turbo
R studio V4.1.1	R-project	https://www.r-project.org/
Fiji V2.14.0	Schindelin et al.^64^	https://fiji.sc/

## Highlights

- Developed SLAM-RT&Tag to profile RNA dynamics within nuclear compartments.
- RNAs persistently associated with compartments, like XIST and MALAT1, are rare.
- Exceptionally long genes are transcribed adjacent to Polycomb domains.
- Incompletely spliced mature mRNAs transiently pass through nuclear speckles.
