# Supplementary material for: Profiling transcriptome composition and dynamics within nuclear compartments using SLAM-RT&Tag
Source: Mol Cell. Author manuscript; Available in PMC 2025 May 5. (PMC12052203; doi:10.1016/j.molcel.2025.02.012)
Supplement: 1 [file NIHMS2063963-supplement-1.pdf]

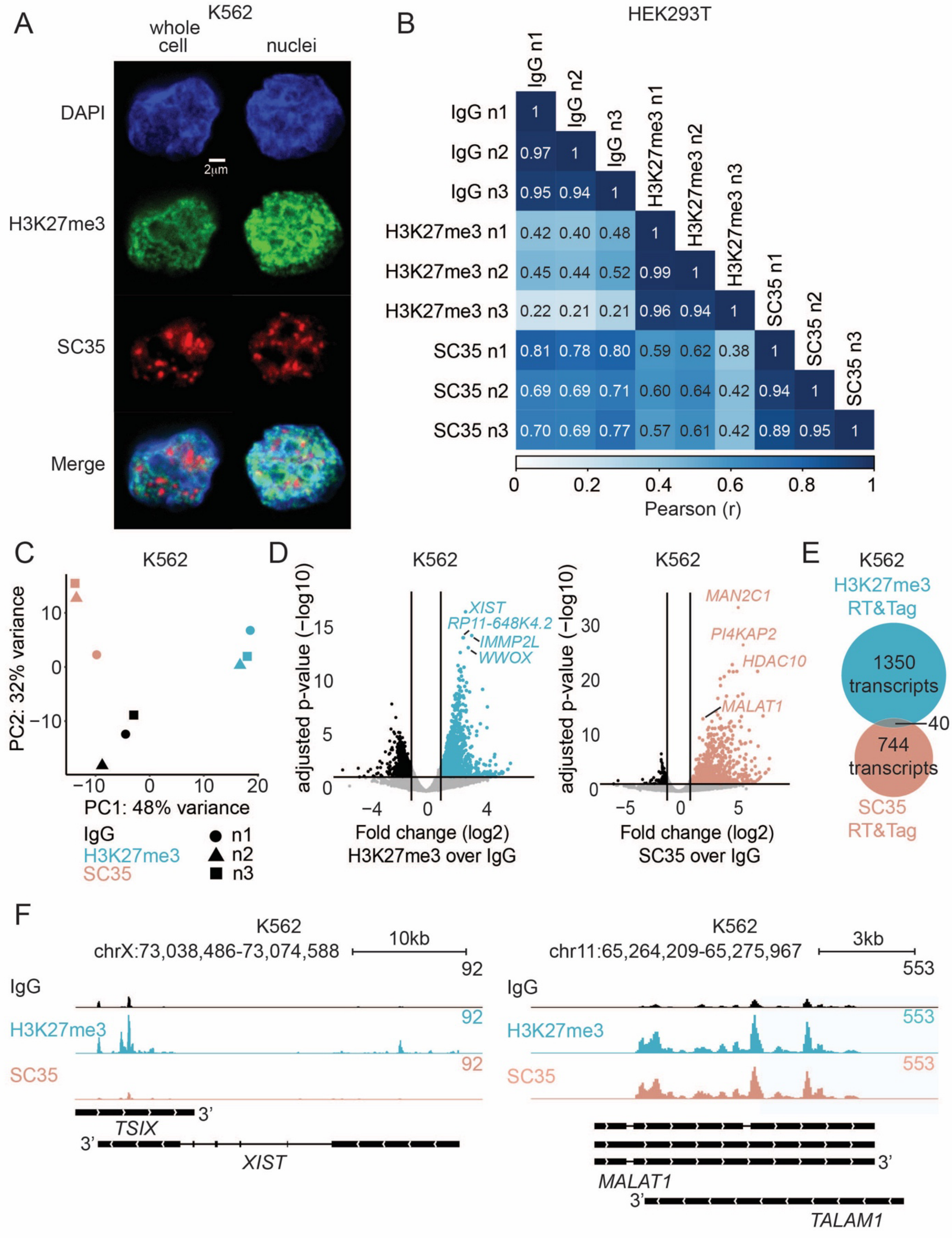

A
K562
whole cell
nuclei
DAPI
H3K27me3
SC35
Merge
2μm
B
HEK293T
IgG n1
IgG n2
IgG n3
H3K27me3 n1
H3K27me3 n2
H3K27me3 n3
SC35 n1
SC35 n2
SC35 n3
Pearson (r)
C
K562
PC1: 48% variance
PC2: 32% variance
IgG
H3K27me3
SC35
n1
n2
n3
D
K562
adjusted p-value (−log10)
XIST
RP11-648K4.2
IMMP2L
WWOX
Fold change (log2)
H3K27me3 over IgG
MAN2C1
PI4KAP2
HDAC10
MALAT1
SC35 over IgG
E
K562
H3K27me3 RT&Tag
1350 transcripts
40
744 transcripts
SC35 RT&Tag
F
K562
chrX:73,038,486-73,074,588
10kb
TSIX
XIST
chr11:65,264,209-65,275,967
3kb
MALAT1
TALAM1

Figure S1

Figure S1. RT&Tag detects RNA within nuclear compartments, related to Figure 1

A) Immunofluorescence images showing DAPI (blue), H3K27me3 (green) and SC35 (red) staining in intact K562 cells and isolated K562 nuclei. Scale bar, 2μm.

B) Correlation matrix displaying Pearson correlation coefficients between 3 biological replicates of IgG, H3K27me3 and SC35-targeted RT&Tag libraries in HEK293T cells.

C) Principal component analysis of IgG, H3K27me3, and SC35-targeted RT&Tag libraries in K562 cells.

D) Volcano plot showing transcripts differentially enriched for H3K27me3 (left, in light blue) and SC35 (right, in salmon) over IgG-targeted RT&Tag in K562 cells (fold change >2, adjusted p-value <0.05, n=3).

E) Venn diagrams showing the overlap in transcripts enriched for H3K27me3 and SC35-targeted RT&Tag in K562 cells.

F) Genome tracks showing IgG, H3K27me3, and SC35-targeted RT&Tag signal over *XIST* (left) and *MALAT1* (right) in K562 cells. Combined alignments from 3 replicates are shown.

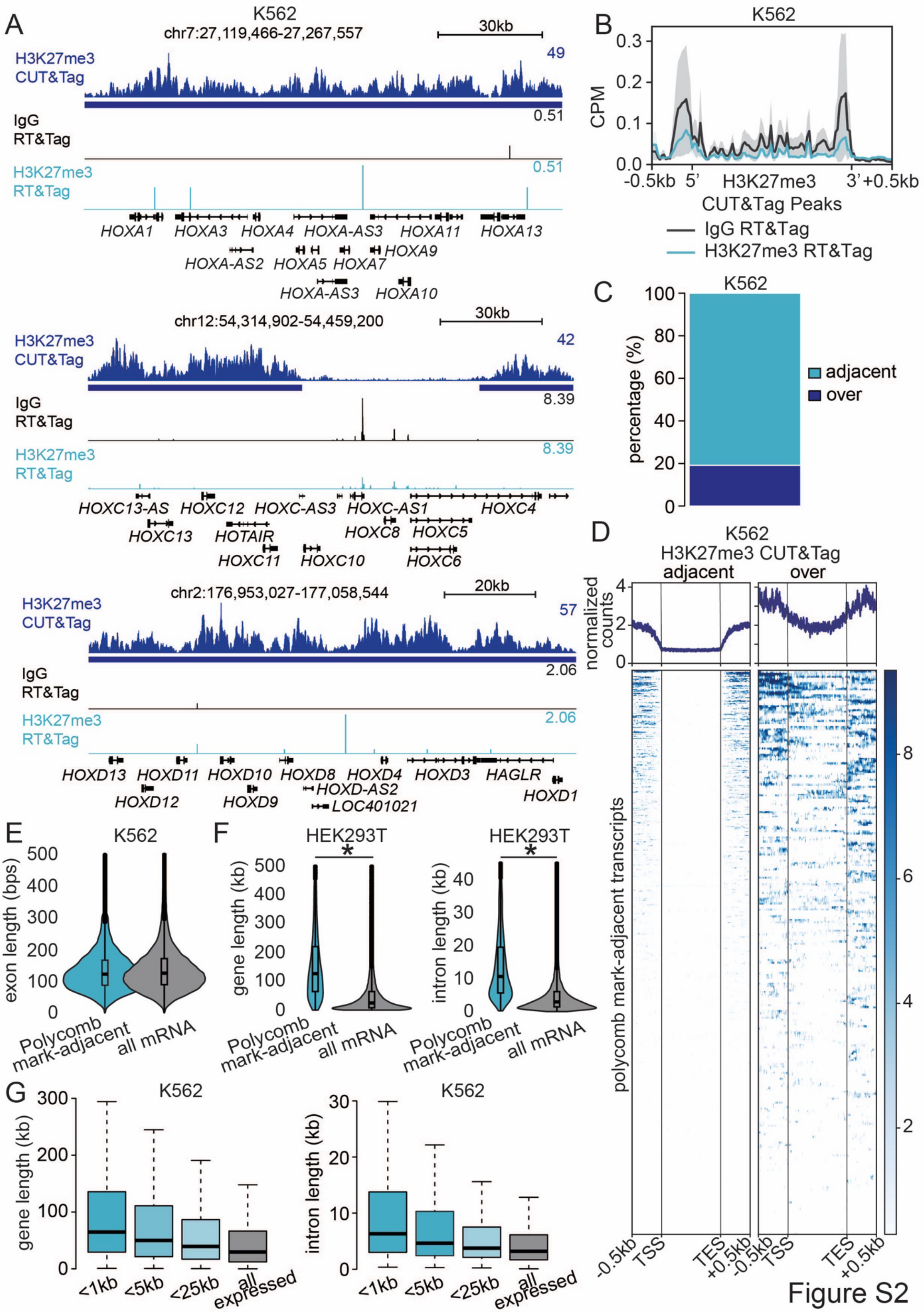

A
K562
chr7:27,119,466-27,267,557
30kb
H3K27me3 CUT&Tag
49
IgG RT&Tag
0.51
H3K27me3 RT&Tag
0.51
HOXA1
HOXA3
HOXA4
HOXA-AS3
HOXA11
HOXA13
HOXA-AS2
HOXA5
HOXA7
HOXA9
HOXA-AS3
HOXA10
chr12:54,314,902-54,459,200
30kb
H3K27me3 CUT&Tag
42
IgG RT&Tag
8.39
H3K27me3 RT&Tag
8.39
HOXC13-AS
HOXC12
HOXC-AS3
HOXC-AS1
HOXC4
HOXC13
HOTAIR
HOXC8
HOXC5
HOXC11
HOXC10
HOXC6
chr2:176,953,027-177,058,544
20kb
H3K27me3 CUT&Tag
57
IgG RT&Tag
2.06
H3K27me3 RT&Tag
2.06
HOXD13
HOXD11
HOXD10
HOXD8
HOXD4
HOXD3
HAGLR
HOXD-AS2
HOXD12
HOXD9
LOC401021
HOXD1
B
K562
CPM
0.3
0.2
0.1
0.0
-0.5kb
5'
H3K27me3
3'
+0.5kb
CUT&Tag Peaks
IgG RT&Tag
H3K27me3 RT&Tag
C
K562
percentage (%)
100
80
60
40
20
0
adjacent
over
D
K562
H3K27me3 CUT&Tag
adjacent
over
normalized counts
4
2
0
polycomb mark-adjacent transcripts
8
6
4
2
-0.5kb
TSS
TES
+0.5kb
-0.5kb
TSS
TES
+0.5kb
E
K562
exon length (bps)
500
400
300
200
100
0
Polycomb mark-adjacent
all mRNA
F
HEK293T
*
gene length (kb)
500
400
300
200
100
0
Polycomb mark-adjacent
all mRNA
HEK293T
*
intron length (kb)
40
30
20
10
0
Polycomb mark-adjacent
all mRNA
G
K562
gene length (kb)
300
200
100
0
<1kb
<5kb
<25kb
all expressed
K562
intron length (kb)
30
20
10
0
<1kb
<5kb
<25kb
all expressed

Figure S2

Figure S2. Long genes are transcribed adjacent to Polycomb domains, related to Figure 2.

A) Genome tracks showing H3K27me3 CUT&Tag signal, H3K27me3 CUT&Tag peaks, along with IgG and H3K27me3-targeted RT&Tag signal over *HOX* genes in K562 cells. Combined alignments from 3 replicates are shown.

B) Profile plot of IgG and H3K27me3-targeted RT&Tag signal over H3K27me3 CUT&Tag peaks in K562 cells. Data are represented as mean ± SEM.

C) Stacked bar graph showing the percentage of Polycomb mark-adjacent transcripts transcribed adjacent or directly over an H3K27me3 CUT&Tag peak in K562 cells.

D) Profile plots and heatmaps of H3K27me3 CUT&Tag signal over the gene bodies of Polycomb mark-adjacent transcripts transcribed adjacent or directly over an H3K27me3 CUT&Tag peak in K562 cells.

E) Violin plots of exon lengths of Polycomb mark-adjacent transcripts and all annotated mRNA transcripts in K562 cells.

F) Violin plots of gene (left) and intron (right) lengths of Polycomb mark-adjacent transcripts and all annotated mRNA transcripts in HEK293T cells. *$p<0.05$, unpaired t-test.

G) Boxplots of gene (left) and intron (right) lengths of genes expressed (>0CPM) in K562 cells within 1kb, 5kb, or 25kb of a Polycomb domain relative to all expressed genes.

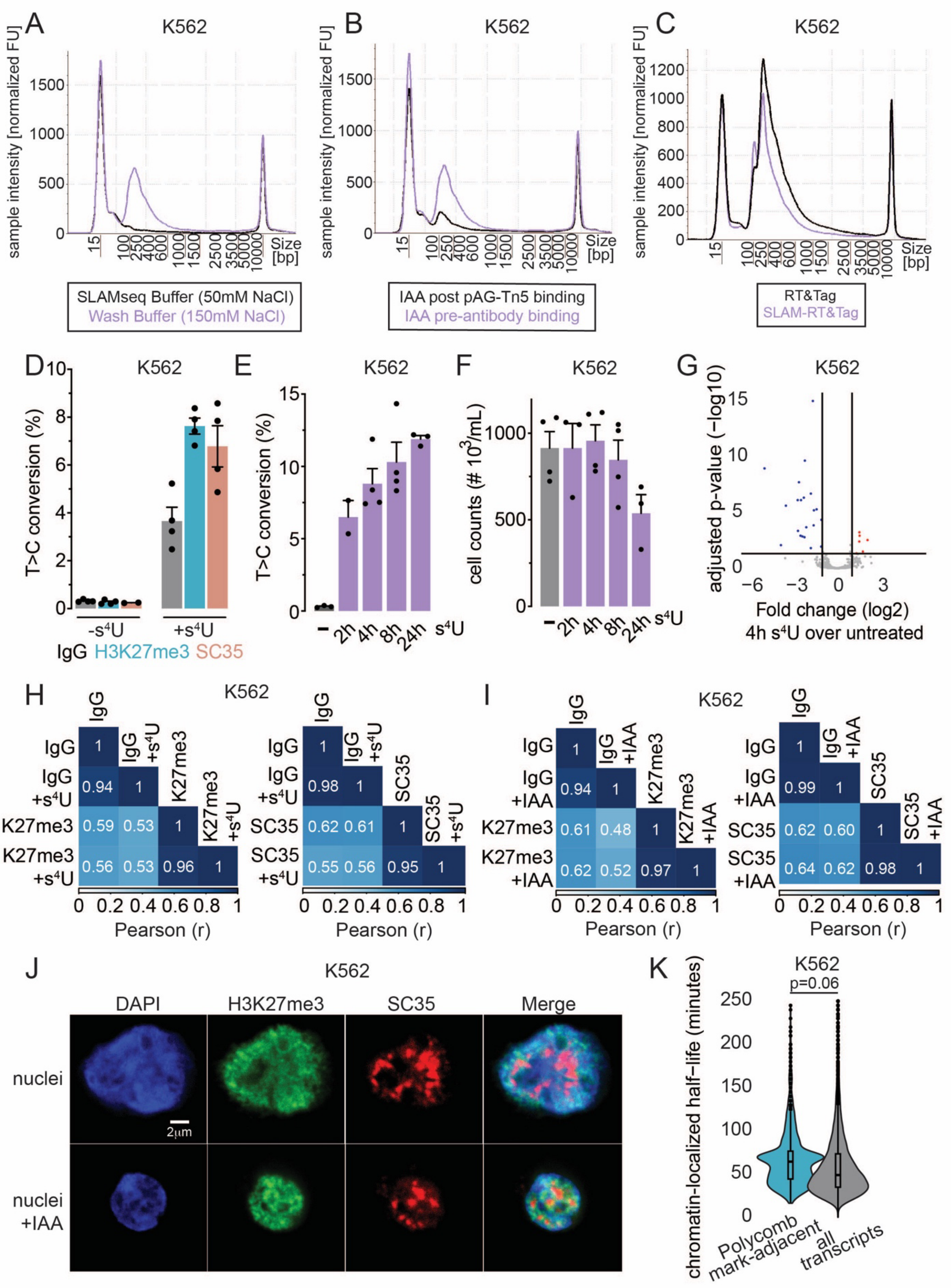

A
K562
sample intensity [normalized FU]
1500
1000
500
0
15
100
250
400
600
1000
1500
2500
3500
5000
10000
Size [bp]
SLAMseq Buffer (50mM NaCl)
Wash Buffer (150mM NaCl)
B
K562
IAA post pAG-Tn5 binding
IAA pre-antibody binding
C
K562
1200
800
400
200
RT&Tag
SLAM-RT&Tag
D
K562
T>C conversion (%)
-s4U
+s4U
IgG H3K27me3 SC35
E
K562
2h
4h
8h
24h
s4U
F
K562
cell counts (# 10^3/mL)
G
K562
adjusted p-value (−log10)
Fold change (log2)
4h s4U over untreated
H
K562
IgG
IgG +s4U
K27me3
K27me3 +s4U
SC35
SC35 +s4U
Pearson (r)
I
K562
IgG +IAA
K27me3 +IAA
SC35 +IAA
J
K562
DAPI
H3K27me3
SC35
Merge
nuclei
nuclei +IAA
2μm
K
K562
p=0.06
chromatin-localized half-life (minutes)
Polycomb mark-adjacent
all transcripts

Figure S3

Figure S3. SLAM-RT&Tag for metabolic labeling of RNA within nuclear compartments, related to Figure 3.

A) Tapestation electropherogram showing the size distribution of H3K27me3-targeted SLAM-RT&Tag libraries in which the iodoacetamide (IAA) reaction was performed in low (50mM) or physiological (150mM) salt wash buffer in K562 cells.

B) Tapestation electropherogram showing the size distribution of H3K27me3-targeted SLAM-RT&Tag libraries in which the IAA reaction was performed before primary antibody binding or after pAG-Tn5 binding in K562 cells.

C) Tapestation electropherogram showing the size distribution of the H3K27me3-targeted SLAM-RT&Tag and RT&Tag libraries in K562 cells.

D) Bar graph of T>C conversion percentages in IgG, H3K27me3 and SC35-targeted SLAM-RT&Tag libraries in K562 cells that were fed or not fed with s^4U. Values from 2-4 biological replicates are plotted as individual dots. Data are represented as mean ± SEM.

E) Bar graph of T>C conversion percentages in H3K27me3-targeted SLAM-RT&Tag libraries in K562 cells fed with s^4U for varying durations. Values from 2-4 biological replicates are plotted as individual dots. Data are represented as mean ± SEM.

F) Bar graph of K562 cell counts at collection following different s^4U feeding durations. Values from 2-4 biological replicates are plotted as individual dots. Data are represented as mean ± SEM.

G) Volcano plot showing differentially expressed genes (>2FC, <0.05p-adj) in K562 cells that were treated with s^4U for 4 hours versus untreated.

H) Correlation matrices showing Pearson correlation coefficients between IgG, H3K27me3 and SC35-targeted RT&Tag libraries in K562 cells that were treated with s^4U for 4 hours versus untreated.

I) Correlation matrices showing Pearson correlation coefficients between IgG, H3K27me3 and SC35-targeted RT&Tag libraries with or without IAA treatment in K562 cells.

J) Immunofluorescence images of DAPI (blue), H3K27me3 (green) and SC35 (red) staining in isolated K562 nuclei that were either untreated (top) or treated with IAA for 1 hour at 37°C (bottom). Scale bar, 2μm.

K) Violin plots of chromatin-localized half-lives reported by Ientswaart et al.[1] for Polycomb mark-adjacent transcripts versus all transcripts in K562 cells. *p<0.05, unpaired t-test.

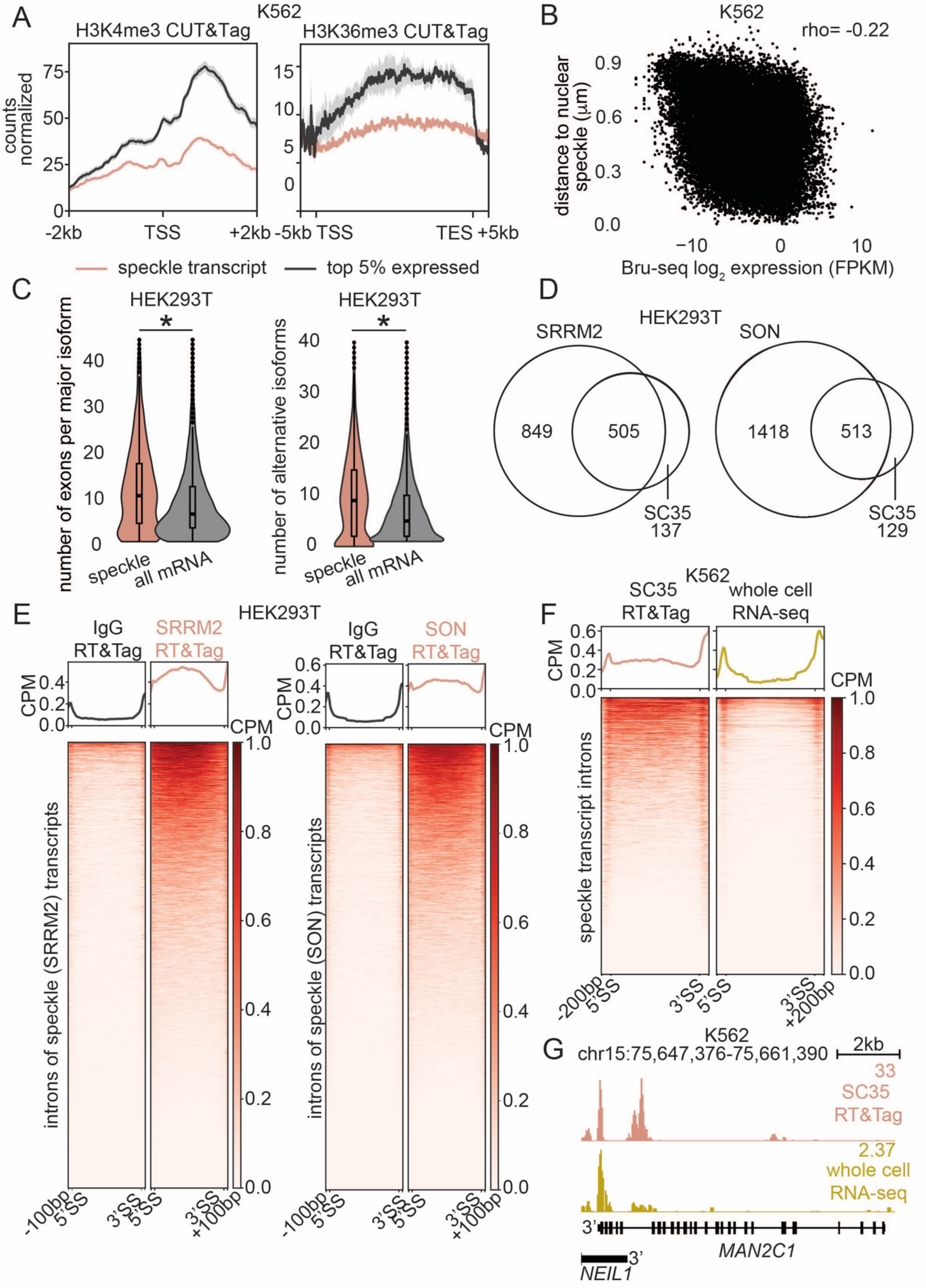
A
K562
H3K4me3 CUT&Tag
H3K36me3 CUT&Tag
normalized counts
-2kb
TSS
+2kb
-5kb TSS
TES +5kb
speckle transcript
top 5% expressed
B
K562
rho= -0.22
distance to nuclear speckle (μm)
Bru-seq log2 expression (FPKM)
C
HEK293T
number of exons per major isoform
number of alternative isoforms
speckle
all mRNA
D
HEK293T
SRRM2
SON
849
505
SC35 137
1418
513
SC35 129
E
HEK293T
IgG RT&Tag
SRRM2 RT&Tag
SON RT&Tag
CPM
introns of speckle (SRRM2) transcripts
introns of speckle (SON) transcripts
-100bp 5'SS
3'SS 5'SS
3'SS +100bp
F
K562
SC35 RT&Tag
whole cell RNA-seq
speckle transcript introns
-200bp 5'SS
3'SS +200bp
G
K562
chr15:75,647,376-75,661,390
2kb
33
SC35 RT&Tag
2.37
whole cell RNA-seq
MAN2C1
NEIL1

Figure S4

Figure S4. Nuclear speckles contain partially spliced polyadenylated transcripts, related to Figure 4.

A) Profile plots of H3K4me3 (top) and H3K36me3 (bottom) CUT&Tag signal at the transcriptional start sites (TSS; top) or over the gene bodies (bottom) of speckle transcripts and the top 5% expressed transcripts in K562 cells. Data are represented as mean ± SEM.

B) Scatter plot showing the lack of correlation between nascent gene expression and distance to the nuclear speckle (μm) measured using TSA-seq in K562 cells. The Spearman correlation coefficient is shown. Each point represents an individual transcript. FPKM- Fragments Per Kilobase of transcript per Million mapped reads.

C) Violin plots showing the number of exons per major isoform (left) and the number of alternative isoforms (right) of speckle transcripts and all annotated mRNA transcripts in HEK293T cells. *$p<0.05$, unpaired t-test.

D) Venn diagrams showing the overlap between SC35-enriched transcripts with SRRM2- (left) or SON- (right) enriched transcripts in HEK293T cells.

E) Heatmaps of IgG, SRRM2, and SON RT&Tag signal over introns of nuclear speckle-associated transcripts detected using the SRRM2 or SON antibodies in HEK293T cells.

F) Heatmaps of SC35-targeted RT&Tag and whole-cell RNA-seq signal over introns of speckle transcripts in K562 cells.

G) Genome track showing SC35-targeted RT&Tag and whole cell RNA-seq signal over the gene body of the speckle transcript *MAN2C1* in K562 cells. Combined reads from 3 replicates are shown.

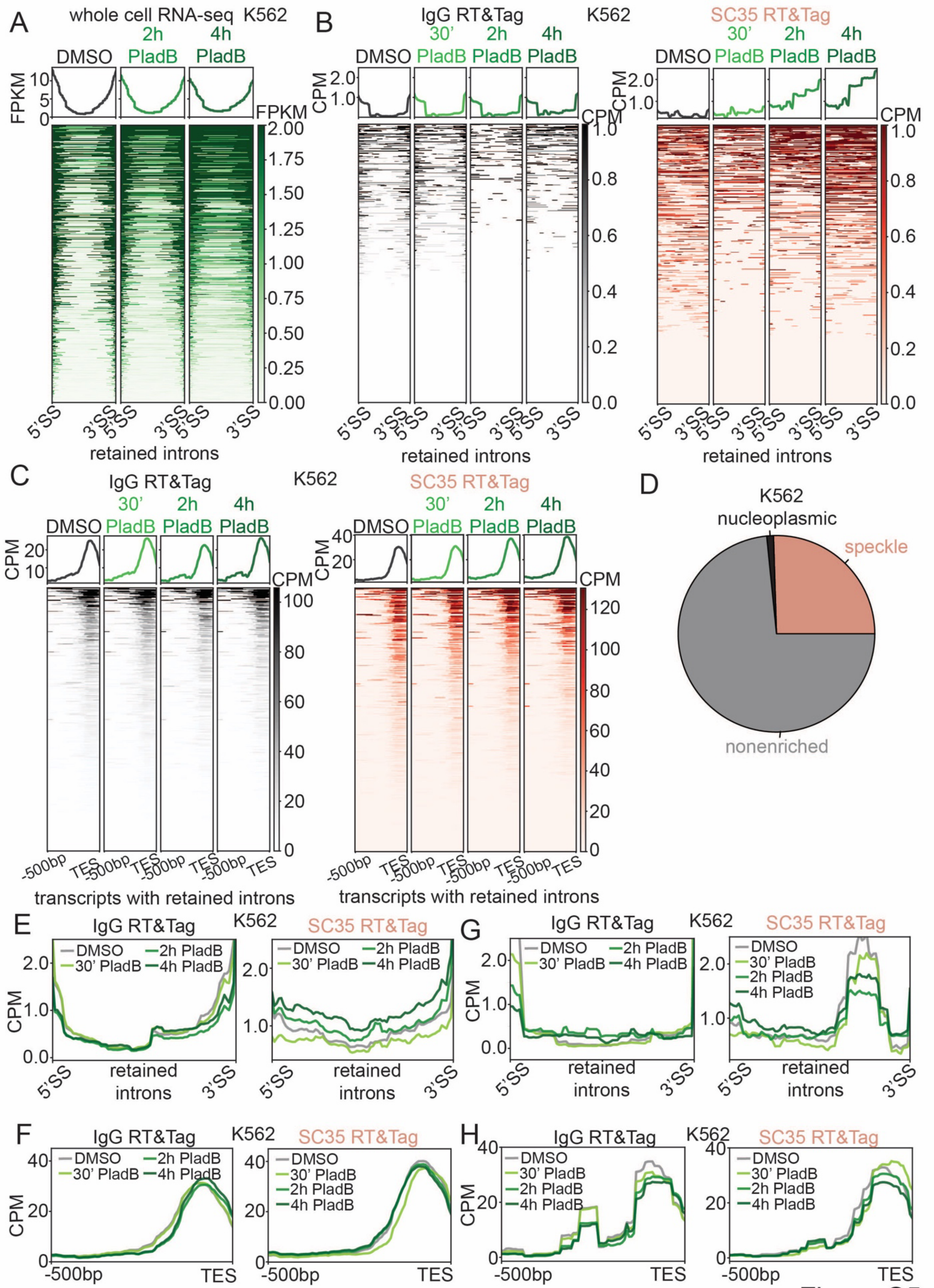
A
whole cell RNA-seq K562
DMSO
2h PladB
4h PladB
FPKM
5'SS
3'SS
retained introns
B
IgG RT&Tag
K562
SC35 RT&Tag
DMSO
30' PladB
2h PladB
4h PladB
CPM
retained introns
C
IgG RT&Tag
K562
SC35 RT&Tag
-500bp
TES
transcripts with retained introns
D
K562
nucleoplasmic
speckle
nonenriched
E
IgG RT&Tag
K562
SC35 RT&Tag
DMSO
30' PladB
2h PladB
4h PladB
retained introns
F
G
H

Figure S5

Figure S5. Incompletely spliced transcripts migrate to nuclear speckles, related to Figure 5.

A) Heatmaps of RNA-seq signal over PladB-induced retained introns in K562 cells treated with PladB for 2 hours and 4 hours. Combined reads from 2 replicates are shown. Heatmap maximum intensity value (zMax) is manually set to 2.

B) Heatmaps of IgG and SC35-targeted RT&Tag signal over PladB-induced retained introns in K562 cells treated with PladB for 30', 2 hours, and 4 hours. Combined reads from 2 replicates are shown. Heatmap maximum intensity value (zMax) is manually set to 1.

C) Heatmaps of IgG and SC35-targeted RT&Tag over the 500bp upstream of the 3'end of transcripts that gain retained introns in K562 cells treated with PladB. Combined reads from 2 replicates are shown.

D) Pie chart showing the fraction of transcripts that retain introns in response to PladB, categorized by their enrichment in nuclear speckles, nucleoplasm, or neither in K562 cells under basal conditions.

E) Profile plots of IgG and SC35-targeted RT&Tag signal over non-retained introns in K562 cells treated with PladB for 30', 2 hours, and 4 hours. Combined reads from 2 replicates are shown.

F) Profile plots of IgG and SC35-targeted RT&Tag signal over the 500bp upstream of the 3'end of transcripts with non-retained in K562 cells treated with PladB. Combined reads from 2 replicates are shown.

G) Profile plots of IgG and SC35-targeted RT&Tag signal over introns that lose retention in K562 cells upon treatment with PladB. Combined reads from 2 replicates are shown.

H) Profile plots of IgG and SC35-targeted RT&Tag signal over the 500bp upstream of the 3'end of transcripts that lose intron retention in K562 cells treated with PladB. Combined reads from 2 replicates are shown.

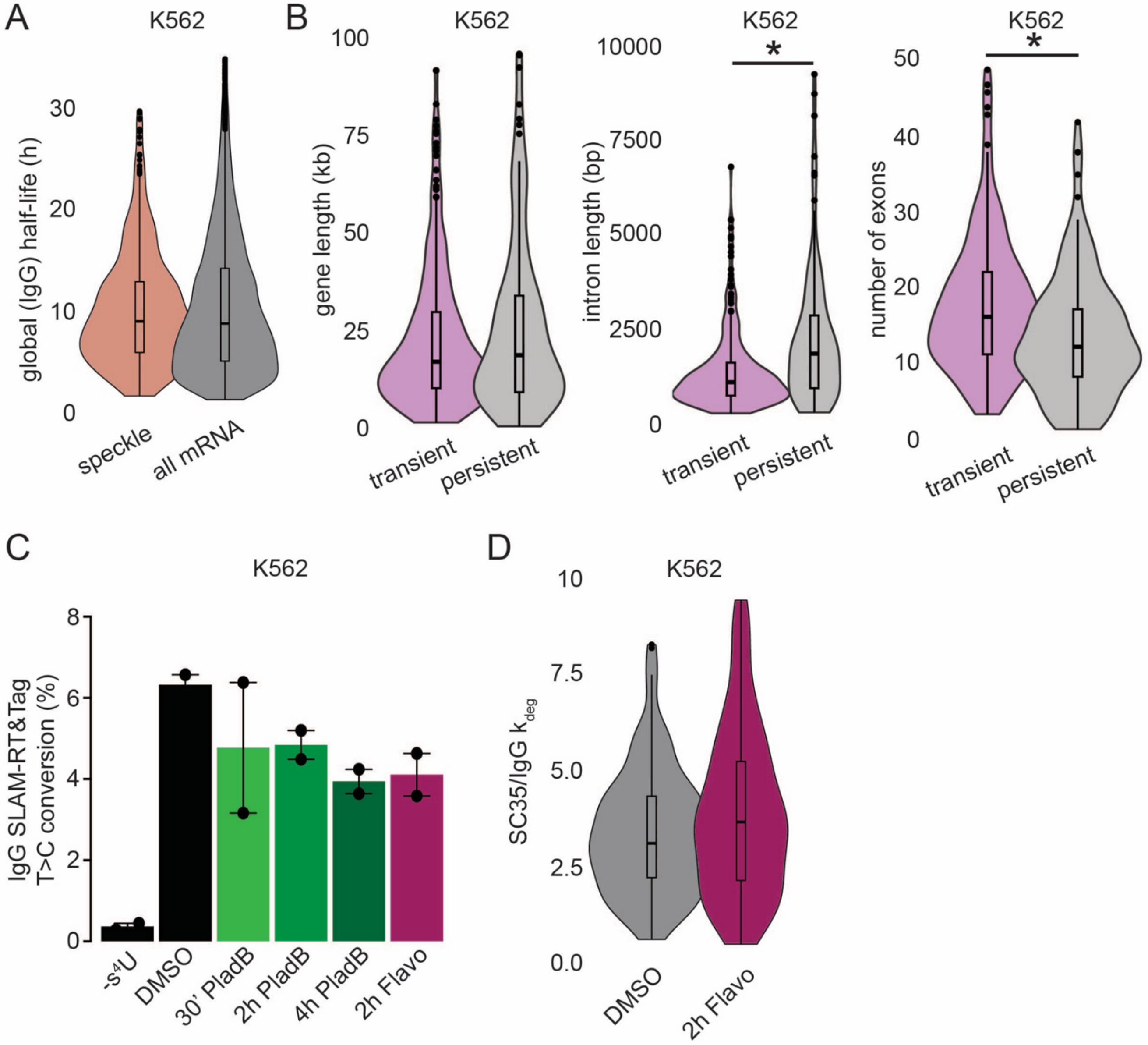
A
K562
global (IgG) half-life (h)
speckle
all mRNA
B
K562
gene length (kb)
transient
persistent
K562
intron length (bp)
*
transient
persistent
K562
number of exons
*
transient
persistent
C
K562
IgG SLAM-RT&Tag T>C conversion (%)
-s4U
DMSO
30' PladB
2h PladB
4h PladB
2h Flavo
D
K562
SC35/IgG k_deg
DMSO
2h Flavo

Figure S6

Figure S6. Speckle transcripts are predominantly transiently withheld in nuclear speckles, related to Figure 6.

A) Violin plots of global (IgG) half-lives of speckle transcripts and all mRNA transcripts in K562 cells.

B) Violin plots of gene lengths (left), intron lengths (middle) and number of exons per major isoform (right) of transient versus persistent speckle transcripts in K562 cells. *$p<0.05$, unpaired t-test.

C) Bar graph of T>C conversion percentages in IgG-targeted SLAM-RT&Tag libraries generated from K562 cells treated with PladB or flavopiridol. Values from 2 biological replicates are plotted as individual dots. Data are represented as mean ± SEM.

D) Violin plots of IgG over SC35 k_{deg} ratio of speckle transcripts in K562 treated with either DMSO or flavopiridol for 2 hours.

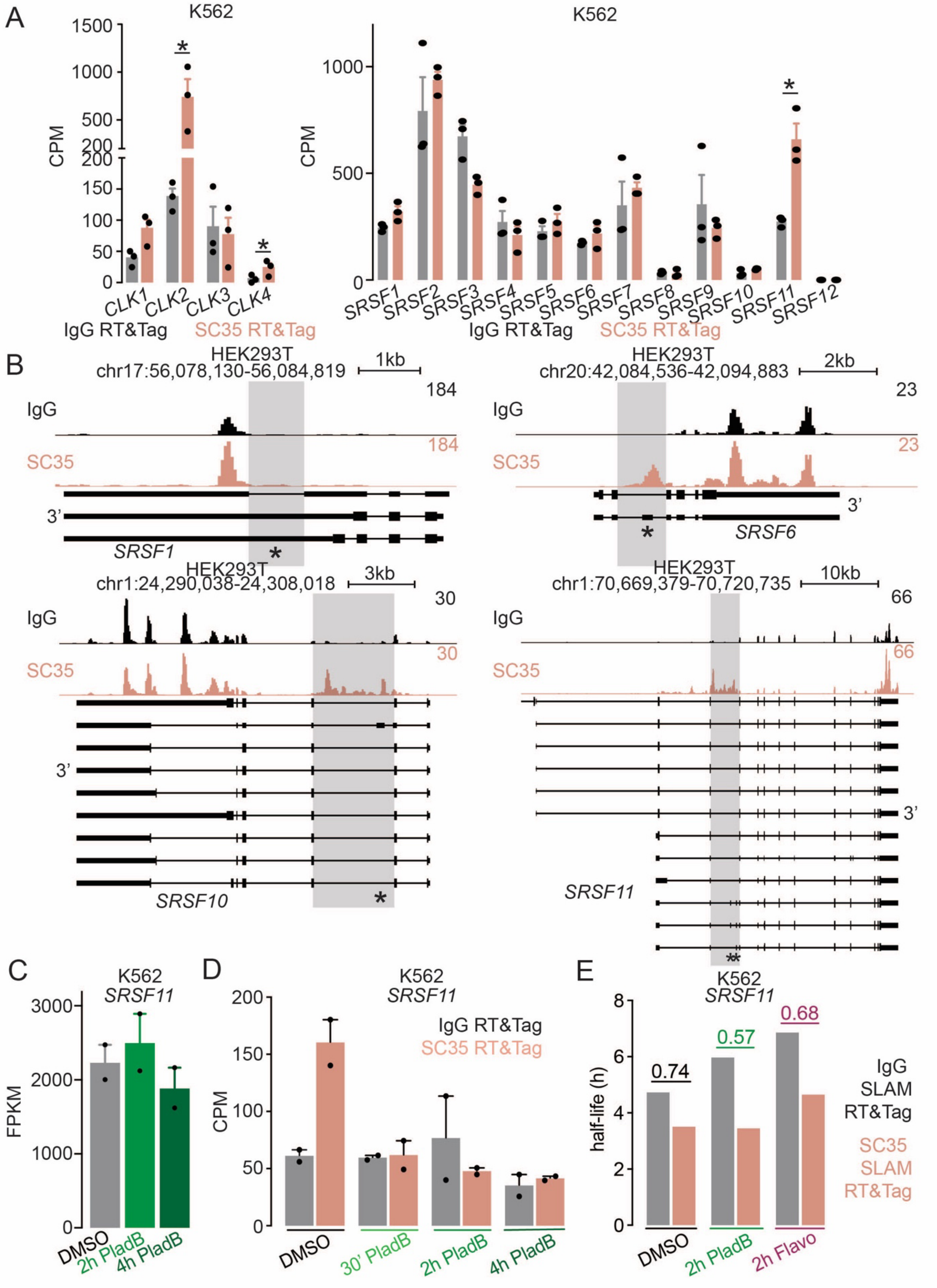

A
K562
CPM
CLK1 CLK2 CLK3 CLK4
IgG RT&Tag
SC35 RT&Tag
SRSF1 SRSF2 SRSF3 SRSF4 SRSF5 SRSF6 SRSF7 SRSF8 SRSF9 SRSF10 SRSF11 SRSF12
B
HEK293T
chr17:56,078,130-56,084,819
1kb
IgG
SC35
SRSF1
chr20:42,084,536-42,094,883
2kb
SRSF6
chr1:24,290,038-24,308,018
3kb
SRSF10
chr1:70,669,379-70,720,735
10kb
SRSF11
C
K562
SRSF11
FPKM
DMSO
2h PladB
4h PladB
D
CPM
30' PladB
E
half-life (h)
0.74
0.57
0.68
IgG SLAM RT&Tag
SC35 SLAM RT&Tag
2h Flavo

Figure S7

Figure S7. *SRSF11* transcripts are rapidly released from nuclear speckles in response to PladB treatment, related to Figure 7.

A) Bar plots of IgG and SC35-targeted RT&Tag counts for *CLK* (left) and *SRSF* (right) transcripts in K562 cells. Individual points represent different biological replicates. Data are represented as mean ± SEM. *$p<0.05$, BH adjusted p-value. CPM- Counts per million.

B) Genome tracks showing IgG and SC35-targeted RT&Tag signal over the gene bodies of *SRSF1*, *SRSF6*, *SRSF10*, and *SRSF11* in HEK293T cells. Poison exons are marked with asterisks and their adjacent introns are highlighted in grey. Combined reads from 3 replicates are shown.

C) Bar plot of whole-cell RNA sequencing counts for *SRSF11* in K562 cells treated with PladB. Individual points represent different biological replicates. Data are represented as mean ± SEM. FPKM- Fragments Per Kilobase of transcript per Million mapped reads.

D) Bar plot of IgG and SC35-targeted RT&Tag counts for *SRSF11* in K562 cells treated with PladB. Individual points represent different biological replicates. Data are represented as mean ± SEM. CPM- Counts per million.

E) Bar plot of global (IgG) and localized (SC35) half-lives of *SRSF11* in K562 cells treated with PladB or flavopiridol. Fold change differences in localized over global half-lives are included.

Supplemental references

1 Ietswaart, R., Smalec, B. M., Xu, A., Choquet, K., McShane, E., Jowhar, Z. M., Guegler, C. K., Baxter-Koenigs, A. R., West, E. R., Fu, B. X. H. *et al.* (2024). Genome-wide quantification of RNA flow across subcellular compartments reveals determinants of the mammalian transcript life cycle. Mol Cell 84, 2765-2784 e2716.
